# Impact of socioeconomic status and country of origin on COVID-19 outcomes in Swedish ICUs: a retrospective registry-based cohort study

**DOI:** 10.1136/bmjopen-2025-099763

**Published:** 2025-11-16

**Authors:** Knut Taxbro, Rasmus Åhman, Michelle S Chew, Lars Engerström

**Affiliations:** 1 Biomedical and Clinical Sciences, Linkoping university, Linköping, Sweden; 2 Anaesthesia and Intensive Care Medicine, Ryhov County Hospital, Jönköping, Sweden; 3 Helicopter Emergency Medical Service, Region Västra Götaland, Gothenburg, Sweden; 4 Department of Anaesthesia and Intensive Care, Biomedical and Clinical Sciences, Linköping University, Linköping, Sweden; 5 Råslätt Primary Health Care Centre, Jönköping, Region Jönköping County, Sweden; 6 Perioperative Medicine and Intensive Care, Karolinska University Hospital, Stockholm, Sweden; 7 Anaesthesia and Intensive Care Medicine, Vrinnevi Hospital in Norrköping, Norrköping, Sweden; 8 Cardiothoracic and Vascular Surgery and Anaesthesia, Linköping University Hospital, Linköping, Sweden

**Keywords:** COVID-19, Intensive Care Units, Mortality

## Abstract

**Abstract:**

**Objectives:**

This study aimed to investigate the impact of socioeconomic status and country of origin on COVID-19 outcomes in Swedish intensive care units (ICUs), hypothesising that these factors are independently associated with 90-day mortality.

**Design:**

Registry-based cohort study.

**Setting:**

Swedish ICUs, from 6 March 2020 to 31 December 2022.

**Participants:**

Adults (≥18 years) with confirmed SARS-CoV-2 infection and acute hypoxaemic respiratory failure. A total of 5833 patients were included in the multivariable model.

**Interventions:**

Not applicable.

**Primary and secondary outcome measures:**

The primary outcome was 90-day mortality. Secondary outcomes included 1-year mortality and ventilator and renal replacement therapy-free days within 60 days post-ICU admission.

**Results:**

Non-European country of origin was independently associated with higher 90-day and 1-year mortality adjusted OR (aOR) 1.34 [95% CI 1.13 to 1.61], p=0.001, aOR 1.26 [95% CI 1.01 to 1.5], p=0.01, respectively. Socioeconomic variables did not significantly impact mortality or organ support-free days. Other predictors of 90-day mortality included age, sex, chronic heart and lung disease, Simplified Acute Physiology Score 3 score, immunosuppression, time in hospital prior to ICU admission, pandemic wave and admission Partial pressure of oxygen in arterial blood/Fraction of inspired oxygen-ratio.

**Conclusions:**

The study identified significant disparities in COVID-19 outcomes based on country of origin, with non-European patients facing higher mortality risks. These findings could challenge the notion of equitable healthcare in Sweden and underscore the need for further research into systemic inequalities.

**STRENGTHS AND LIMITATIONS OF THIS STUDY**

- Comprehensive data from the Swedish Intensive Care Registry.
- Robust adjustments for age, sex, chronic diseases, severity of illness and vaccination status.
- Nationwide scope.
- Inability to identify second-generation immigrants.
- Lack of a deprivation index in adjustments.

## Introduction

 Sweden’s healthcare system is noteworthy for being almost entirely publicly funded and providing universal coverage. Swedish legislation mandates equitable access to healthcare regardless of socioeconomic status or ethnicity. Therefore, one might assume that there are only minor disparities in treatment and outcomes for patients receiving medical care in Sweden.

Globally, persons belonging to ethnic minorities have been shown to bear a greater disease burden and have a higher risk for hospitalisation, ICU admission and death during the COVID-19 pandemic.^1^

Foreign-born workers in essential occupations in Sweden were found to have a higher risk of hospitalisation and intensive care unit (ICU) admission due to COVID-19.^2^

Furthermore, individuals with an immigrant background, lower levels of education and lower income were more likely to experience severe COVID-19 requiring mechanical ventilation during the pandemic’s first 15 months. Specifically, being born in Africa and having a lower income level were associated with increased 90-day mortality. Whether this reflects higher infection rates in these groups or a worse prognosis after hospital admission remains unclear. Patient-related factors such as comorbidities, lifestyle characteristics or delayed presentation may also contribute to poorer outcomes despite equal access to care.^3^ Nevertheless, these findings challenge the concept of equitable healthcare in Sweden. National cohort studies focusing on patients admitted to ICUs analysing treatment and outcomes across various socioeconomic and country-of-origin domains are scarce.

Therefore, we aimed to examine the characteristics, management and mortality outcomes following ICU admissions caused by COVID-19 with acute hypoxemic respiratory failure across various socioeconomic and country-of-origin groups in Sweden. Using high-quality individual-level data from linked national registries, we hypothesised that socioeconomic status and country of origin were independently associated with 90-day mortality.

## Methods

We conducted a registry-based cohort study of all adult (≥18 years of age) patients with a Swedish personal identity number admitted to Swedish ICUs with PCR confirmed SARS-CoV-2 infection and acute hypoxaemic respiratory failure due to COVID-19 disease (ICD-10-SE diagnosis code U07.1) between 6 March 2020 and 31 December 2022. We extracted individual data by combining the Swedish Intensive Care Registry (SIR) and Statistics Sweden on a national level. Patients with incomplete 90-day mortality data were excluded from the final analysis. The study is reported in accordance with Strengthening the Reporting of Observational Studies (STROBE).^4^

### Study objectives

We aimed to study the association between socioeconomic factors and country-of-origin (exposure) and death at 90 days (primary outcome), 1 year (secondary outcome) and ventilator and renal replacement therapy (RRT)-free days (secondary outcome) within 60 days after the index ICU admission after adjusting for potential confounders. The exposures were socioeconomic factors and country of origin. The primary outcome was 90-day all-cause mortality. Secondary outcomes were 1-year mortality and the number of days alive and free from organ support (ventilator and/or RRT) within 60 days following the index ICU admission. Patients who died within 60 days were assigned zero organ support-free days, and follow-up was censored at 60 days.

### Definitions

Socioeconomic status was defined as level of income reported to the Swedish Tax Agency and was divided into quartiles based on the 2019 fiscal year, level of education was categorised based on the highest attained level (elementary school drop-out, complete elementary school, any upper secondary school, university (<9, 9, >9–12 or ≥13 years, respectively)), marital status (registered or no registered partner) and household size (1 or >1 person). Since data on ethnicity are not registered, this exposure was represented by its surrogate and country of origin and grouped using the United Nations^5^ classification (Nordic, Europe and other (Asia, the Americas, Africa and Oceania)). Population density was classified as rural or urban.^6^ These data were extracted from Statistics Sweden after data linkage with index patients identified in the SIR.

Chronic heart, lung, renal, hepatic and neuromuscular diseases were extracted from the SIR as at the discretion of the treating physician. Acute respiratory distress syndrome (ARDS) was defined using the ICD codes (‘J809’, ‘J809A’, ‘J809B’, ‘J809C’ and ‘J809X’).

The five pandemic waves were defined according to the Swedish National Board of Health and Welfare to the following time periods: Wave 1 (01-03-2020 to 30-09-2020), Wave 2 (01-10-2020 to 31-01- 2021), Wave 3 (01-02-2021 to 30-06-2021), Wave 4 (01-07-2021 to 31-12-2021) and Wave 5 (01-01-2022 to 31-12-2022).^7^

Daily data regarding ICU management and ICU admission characteristics are prospectively registered into the SIR database by end users. The variables include treatment with continuous RRT, severity of ARDS (none, mild, moderate and severe), level of respiratory support (high-flow nasal oxygenation, non-invasive ventilation, invasive mechanical ventilation (IMV) and prone position ventilation), COVID-specific pharmacotherapy (antivirals, steroids, Interleukin-6 and Januskinase inhibitors), vasoactive pharmacotherapy, transfer to another ICU, time from ICU admission to IMV, time in IMV and ICU length of stay and hospital length of stay prior to ICU admission. These variables are defined according to SIR’s ‘Standard Operating Procedures for registration’. More information is available at the SIR website.^8^

The study was approved by the Swedish Ethical Review Authority (reference number 2022-04801-02). Patients and/or their next-of-kin were informed of the registry and given the possibility to opt out at any time. Patient data integrity is ensured by anonymisation.

### Patient and public involvement

No patients or members of the public were involved in the design, conduct or reporting of this research. A summary of the key findings will be disseminated to the public on publication of the final manuscript.

### Statistical analysis

Continuous variables are described using medians and IQR. Categorical variables are given as frequencies and percentages. We used t-tests, Mann-Whitney U tests, χ^2^ or Fisher’s exact tests for comparisons of survivors versus non-survivors.

A multivariable model was used to determine the independent association between predictor variables and the primary and secondary outcomes. Logistic regression was used to assess the independent effect (adjusted OR) of the two main exposures and 90-day mortality. No imputation was conducted. The predictor variables were chosen a priori based on prior knowledge and clinical plausibility. Variables with p<0.10 in univariable analyses were included in the multivariable models. Analyses were performed on complete cases, resulting in varying sample sizes across models.

An ordinal regression model was used to analyse the association between organ support-free days (ventilator or RRT by themselves or in combination) within 60 days (secondary outcomes). Cox regression survival plots adjusted for significant covariates were used to assess the association between country of origin and mortality within 1 year after the first ICU admission.

We performed three ancillary analyses: a sensitivity analysis stratifying patients by age (under 65 vs 65 and older), a subgroup analysis limited to patients on IMV and a general estimating equations model to account for clustering by the admitting hospital. E-values were calculated, which estimate how strong an unmeasured confounder would need to explain away an observed association between the exposure and outcome. The higher the E-value, the more robust the association is to potential unmeasured confounding. Conversely, a small E-value suggests the observed association could plausibly be due to an unmeasured confounder.

## Results

**Figure 1:**
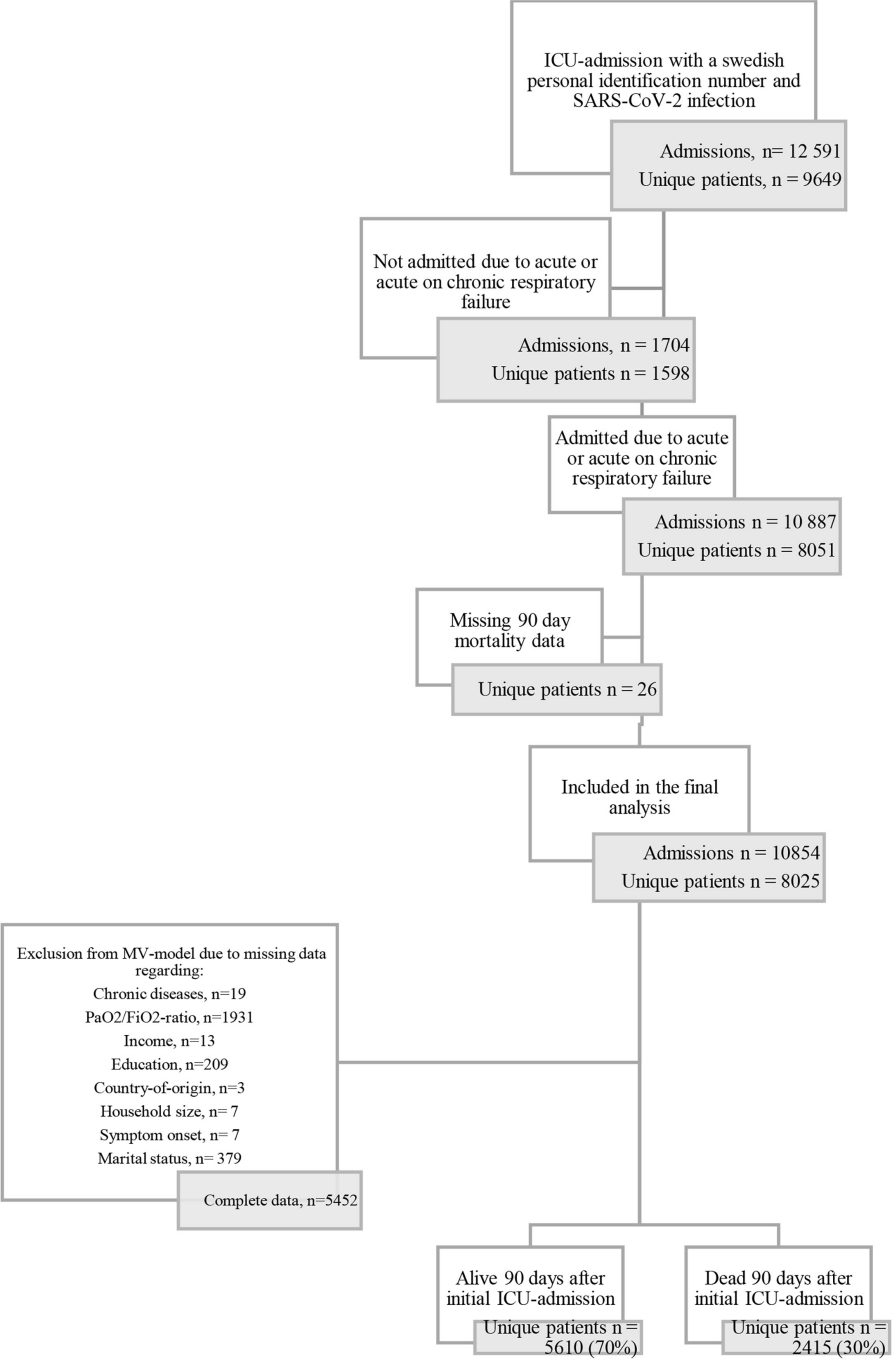
Study outline. Admissions from 6 March 2020 to 31 December 2022. ICU, intensive care unit; MV, mechanical ventilation.

Complete follow-up data regarding 90-day mortality were available for 10 854 ICU admissions of 8025 unique patients between 6 March 2020 and 31 December 2022 (figure 1). The mean follow-up time was 978 days. The median age was 63 years (54.0, 72.0), and the majority of patients were men (70.3%). A majority were born in a Nordic country (64.6%), and the most prevalent non-Nordic country-of-origin group was Asian (16.5%). The unadjusted 90-day mortality was 30.1% for the entire cohort. Within the groups Nordic, European and non-European country of origin, the crude mortality rates were 32.0%, 27.2% and 26.2%, respectively. Data on socioeconomic status are reported in table 1, along with demographic and comorbidity data among survivors and non-survivors. Fifty-seven percent of patients were diagnosed as having severe ARDS, and 70% were treated with IMV for a median of 11 (5.9, 19.8) days. Treatment and ICU characteristics are shown in table 2. Univariable and multivariable logistic regression analyses for 90-day and 1-year mortality are shown in table 3 and online supplemental file 1, respectively.

**Table 1:** Baseline characteristics of 90-day survivors versus non-survivors

Variable	Overall	Survivors	Non-survivors	P	% missing
No.	8025	5610	2415		
Age (median (IQR))	64.0 (54.0, 72.0)	60.0 (51.0, 69.0)	71.0 (63.0, 76.5)	<0.001	0.0
Age group (%)				<0.001	0.0
<50	1381 (17.2)	1268 (22.6)	113 (4.7)		
50–59	1700 (21.2)	1409 (25.1)	291 (12.0)		
60–69	2322 (28.9)	1649 (29.4)	673 (27.9)		
70–79	2130 (26.5)	1103 (19.7)	1027 (42.5)		
80+	492 (6.1)	181 (3.2)	311 (12.9)		
Age>65 (%)	3800 (47.4)	2055 (36.6)	1745 (72.3)	<0.001	0.0
Male sex (%)	5650 (70.4)	3897 (69.5)	1753 (72.6)	0.005	0.0
Body mass index (mean (SD))	30.6 (7.0)	31.2 (7.0)	29.2 (6.9)	<0.001	64.2
SAPS 3 score (median (IQR))	55.0 (49.0, 62.0)	53.0 (47.0, 59.0)	61.0 (55.0, 69.0)	<0.001	0.0
Hypertension (%)	3620 (45.2)	2339 (41.8)	1281 (53.2)	<0.001	0.2
Chronic heart disease (%)	1330 (16.6)	698 (12.5)	632 (26.3)	<0.001	0.2
Chronic lung disease (%)	1419 (17.7)	871 (15.6)	548 (22.8)	<0.001	0.2
Severe obesity (%)	691 (8.6)	530 (9.5)	161 (6.7)	<0.001	0.2
Diabetes mellitus (%)	2088 (26.1)	1358 (24.3)	730 (30.3)	<0.001	0.2
Chronic renal disease (%)	516 (6.4)	273 (4.9)	243 (10.1)	<0.001	0.2
Chronic hepatic disease (%)	77 (1.0)	40 (0.7)	37 (1.5)	0.001	0.2
Chronic neuromuscular disease (%)	152 (1.9)	93 (1.7)	59 (2.5)	0.022	0.2
Immunosuppression (%)	746 (9.3)	410 (7.3)	336 (14.0)	<0.001	0.2
COVID-19 vaccination status (%)				<0.001	0.0
No	6471 (80.6)	4629 (82.5)	1842 (76.3)		
Partly	257 (3.2)	157 (2.8)	100 (4.1)		
Full	450 (5.6)	248 (4.4)	202 (8.4)		
Unknown	847 (10.6)	576 (10.3)	271 (11.2)		
PaO_2_/FiO _2_ (mean (SD))	14.2 (9.5)	14.6 (10.1)	13.1 (7.9)	<0.001	24.1
Time in hospital prior to ICU admission, days (median (IQR))	2.0 (0.0, 4.0)	1.0 (0.0, 4.0)	2.0 (0.0, 6.0)	<0.001	0.0
Symptom onset to ICU, days (median (IQR))	10.5 (7.7, 13.5)	10.5 (7.9, 13.3)	10.2 (7.2, 14.1)	0.068	0.3
Pandemic wave (%)				<0.001	0.0
Wave 1	2290 (28.5)	1664 (29.7)	626 (25.9)		
Wave 2	1943 (24.2)	1285 (22.9)	658 (27.2)		
Wave 3	2484 (31.0)	1807 (32.2)	677 (28.0)		
Wave 4	578 (7.2)	431 (7.7)	147 (6.1)		
Wave 5	730 (9.1)	423 (7.5)	307 (12.7)		
Income quartiles (%)				<0.001	0.2
Q1	1946 (24.3)	1322 (23.6)	624 (26.0)		
Q2	1907 (23.8)	1148 (20.5)	759 (31.6)		
Q3	2012 (25.1)	1443 (25.7)	569 (23.7)		
Q4	2145 (26.8)	1693 (30.2)	452 (18.8)		
Education level (%)				<0.001	3.6
<9 years	1215 (15.7)	711 (13.1)	504 (21.8)		
>12 years	2017 (26.1)	1458 (26.9)	559 (24.2)		
10–12 years	3463 (44.7)	2496 (46.0)	967 (41.8)		
9 years	1045 (13.5)	762 (14.0)	283 (12.2)		
Country of origin (%)				<0.001	0.0
European	952 (11.9)	693 (12.4)	259 (10.7)		
Nordic	5181 (64.6)	3521 (62.8)	1660 (68.8)		
Other	1889 (23.5)	1395 (24.9)	494 (20.5)		
Multiperson household (%)	5886 (73.6)	4244 (75.9)	1642 (68.4)	<0.001	0.4
Registered partner (%)	4222 (56.5)	2957 (55.9)	1265 (57.9)	0.117	6.8
Urban (%)	7234 (90.5)	5045 (90.2)	2189 (91.2)	0.144	0.4

Numbers are No. (%) unless otherwise noted. Global p values for categorical variables were calculated using likelihood ratio tests.

SAPS 3, Simplified Acute Physiology Score 3.

**Table 2:** Treatments of 90-day survivors versus non-survivors

Variable	Overall	Survivors	Non-survivors	P	% missing
No.	8025	5610	2415		
ARDS level (%)				<0.001	24.9
Mild	360 (6.0)	298 (7.2)	62 (3.3)		
Moderate	2258 (37.5)	1858 (44.8)	400 (21.3)		
Severe	3411 (56.6)	1992 (48.0)	1419 (75.4)		
Highest level of respiratory support (%)				<0.001	5.7
HFNO	914 (12.1)	795 (15.2)	119 (5.1)		
NIV	1391 (18.4)	1029 (19.7)	362 (15.5)		
IMV	5260 (69.5)	3407 (65.1)	1853 (79.4)		
On IMV (%)	5260 (65.5)	3407 (60.7)	1853 (76.7)	<0.001	0.0
Days in IMV (median (IQR))	11.0 (5.9, 19.8)	10.3 (5.7, 19.2)	12.7 (6.4, 20.7)	<0.001	0.0
Time from ICU admission to IMV (median (IQR))	5.2 (0.8, 28.2)	5.0 (0.8, 25.0)	6.3 (0.8, 41.8)	0.001	0.0
IMV within 24 hours (%)	3769 (47.0)	2518 (44.9)	1251 (51.8)	<0.001	0.0
Prone position ventilation (%)	3756 (49.6)	2478 (46.8)	1278 (56.1)	<0.001	5.7
CRRT (%)	943 (12.9)	439 (8.7)	504 (22.4)	<0.001	8.7
Remdesivir treatment (%)	948 (11.8)	650 (11.6)	298 (12.4)	0.230	0.2
Lopinavir or ritonavir treatment (%)	8 (0.1)	6 (0.1)	2 (0.1)	1.000	0.2
Baricitinib treatment (%)	50 (0.6)	31 (0.6)	19 (0.8)	0.219	0.2
Steroid treatment (%)	5225 (65.3)	3654 (65.2)	1571 (65.3)	0.958	0.2
IL-6 inhibitor treatment (%)	654 (8.2)	508 (9.1)	146 (6.1)	<0.001	0.2
ICU length of stay (median (IQR))	9.6 (4.0, 18.3)	8.7 (3.9, 17.1)	11.9 (4.6, 20.1)	<0.001	0.0
Multiple ICU admissions (%)	2135 (26.6)	1536 (27.4)	599 (24.8)	0.018	0.0
Transfer to other ICU (%)	1892 (23.6)	1361 (24.3)	531 (22.0)	0.030	0.0
Transfers (%)				0.035	0.0
None	6133 (76.4)	4249 (75.7)	1884 (78.0)		
One	1394 (17.4)	991 (17.7)	403 (16.7)		
Two	407 (5.1)	297 (5.3)	110 (4.6)		
Three or more	91 (1.1)	73 (1.3)	18 (0.7)		
Vasoactive drug treatment (%)	1923 (71.3)	1221 (65.9)	702 (83.3)	<0.001	66.4

Numbers are No. (%) unless otherwise noted. ARDS level as diagnosed during ICU stay.

ARDS, acute respiratory distress syndrome; CRRT, continuous renal replacement therapy; HFNO, high-flow nasal oxygenation; ICU, intensive care unit; IMV, invasive mechanical ventilation; NIV, non-invasive ventilation.

**Table 3:** Univariable and multivariable regression analysis of 90-day mortality. After excluding variables with p>0.1 in the univariable analysis which had some missing data, 5452 patients were included in the multivariable analysis. Hosmer-Lemeshow goodness of fit test did not show a significant deviation from perfect calibration in 10 equally sized groups, p=0.75

Covariate	Unadjusted OR (95% CI)	P	Adjusted OR (95% CI)	P (adj)
Age group		<0.001		<0.001
<50	Reference		Reference	
50–59	2.32 (1.85, 2.93)	<0.001	2.54 (1.90, 3.38)	<0.001
60–69	4.58 (3.72, 5.69)	<0.001	3.45 (2.61, 4.55)	<0.001
70–79	10.45 (8.50, 12.95)	<0.001	5.56 (4.15, 7.46)	<0.001
80+	19.28 (14.83, 25.24)	<0.001	9.80 (6.75, 14.24)	<0.001
Sex		0.005		<0.001
Female	Reference		Reference	
Male	1.16 (1.05, 1.29)		1.30 (1.12, 1.51)	
SAPS 3 score	1.09 (1.08, 1.09)	<0.001	1.06 (1.05, 1.07)	<0.001
Hypertension	1.59 (1.44, 1.75)	<0.001	0.90 (0.79, 1.03)	0.14
Chronic heart disease	2.50 (2.22, 2.82)	<0.001	1.30 (1.10, 1.53)	0.002
Chronic lung disease	1.60 (1.42, 1.80)	<0.001	1.50 (1.28, 1.76)	<0.001
Severe obesity	0.69 (0.57, 0.82)	<0.001	1.13 (0.89, 1.44)	0.31
Diabetes mellitus	1.36 (1.22, 1.51)	<0.001	1.09 (0.94, 1.26)	0.25
Chronic renal disease	2.19 (1.83, 2.62)	<0.001	1.11 (0.87, 1.44)	0.41
Chronic hepatic disease	2.17 (1.38, 3.40)	<0.001	0.83 (0.44, 1.57)	0.57
Chronic neuromuscular disease	1.49 (1.06, 2.06)	0.018	1.25 (0.77, 2.03)	0.37
Immunosuppression	2.05 (1.76, 2.39)	<0.001	1.52 (1.23, 1.87)	<0.001
COVID-19 vaccination		<0.001		0.49
No	Reference		Reference	
Partly	1.60 (1.24, 2.06)	<0.001	1.14 (0.80, 1.62)	0.48
Full	2.05 (1.69, 2.48)	<0.001	0.93 (0.67, 1.29)	0.66
Unknown	1.18 (1.01, 1.38)	0.033	1.15 (0.92, 1.44)	0.23
PaO_2_/FiO_2_	0.98 (0.97, 0.99)	<0.001	0.98 (0.97, 0.99)	<0.001
Spent time in hospital prior to ICU admission	1.06 (1.05, 1.07)	<0.001	1.03 (1.01, 1.04)	<0.001
Symptom onset prior to ICU admission	1.01 (1.00, 1.01)	0.07	0.99 (0.98, 1.01)	0.32
Wave		<0.001		0.086
Wave 1	Reference		Reference	
Wave 2	1.36 (1.19, 1.55)	<0.001	0.82 (0.69, 0.98)	0.029
Wave 3	1.00 (0.88, 1.13)	0.95	0.79 (0.66, 0.95)	0.014
Wave 4	0.91 (0.73, 1.11)	0.36	0.87 (0.64, 1.18)	0.37
Wave 5	1.93 (1.62, 2.29)	<0.001	0.75 (0.56, 1.00)	0.054
Income quartiles		<0.001		0.25
Q4	Reference		Reference	
Q1	1.77 (1.54, 2.04)	<0.001	1.16 (0.94, 1.43)	0.17
Q2	2.48 (2.16, 2.85)	<0.001	1.21 (1.00, 1.47)	0.051
Q3	1.48 (1.28, 1.70)	<0.001	1.15 (0.96, 1.38)	0.13
Education level		<0.001		0.57
>12 years	Reference		Reference	
<9 years	1.85 (1.59, 2.15)	<0.001	1.07 (0.87, 1.31)	0.52
10–12 years	1.01 (0.89, 1.14)	0.87	0.95 (0.81, 1.12)	0.54
9 years	0.97 (0.82, 1.14)	0.71	0.92 (0.74, 1.15)	0.47
Country of origin		<0.001		0.005
Nordic	Reference		Reference	
European	0.79 (0.68, 0.92)	0.003	1.08 (0.88, 1.33)	0.47
Other	0.75 (0.67, 0.84)	<0.001	1.34 (1.13, 1.61)	0.001
Household size		<0.001		0.47
Single-person household	Reference		Reference	
Multiperson household	0.69 (0.62, 0.77)		0.95 (0.83, 1.10)	
Marital status		0.11		
No registered partner	Reference			
Registered partner	1.09 (0.98, 1.20)			
Population density		0.13		
Rural	Reference			
Urban	1.14 (0.96, 1.34)			

adj, adjusted; ICU, intensive care unit; SAPS 3, Simplified Acute Physiology Score 3.

**Figure 2:**
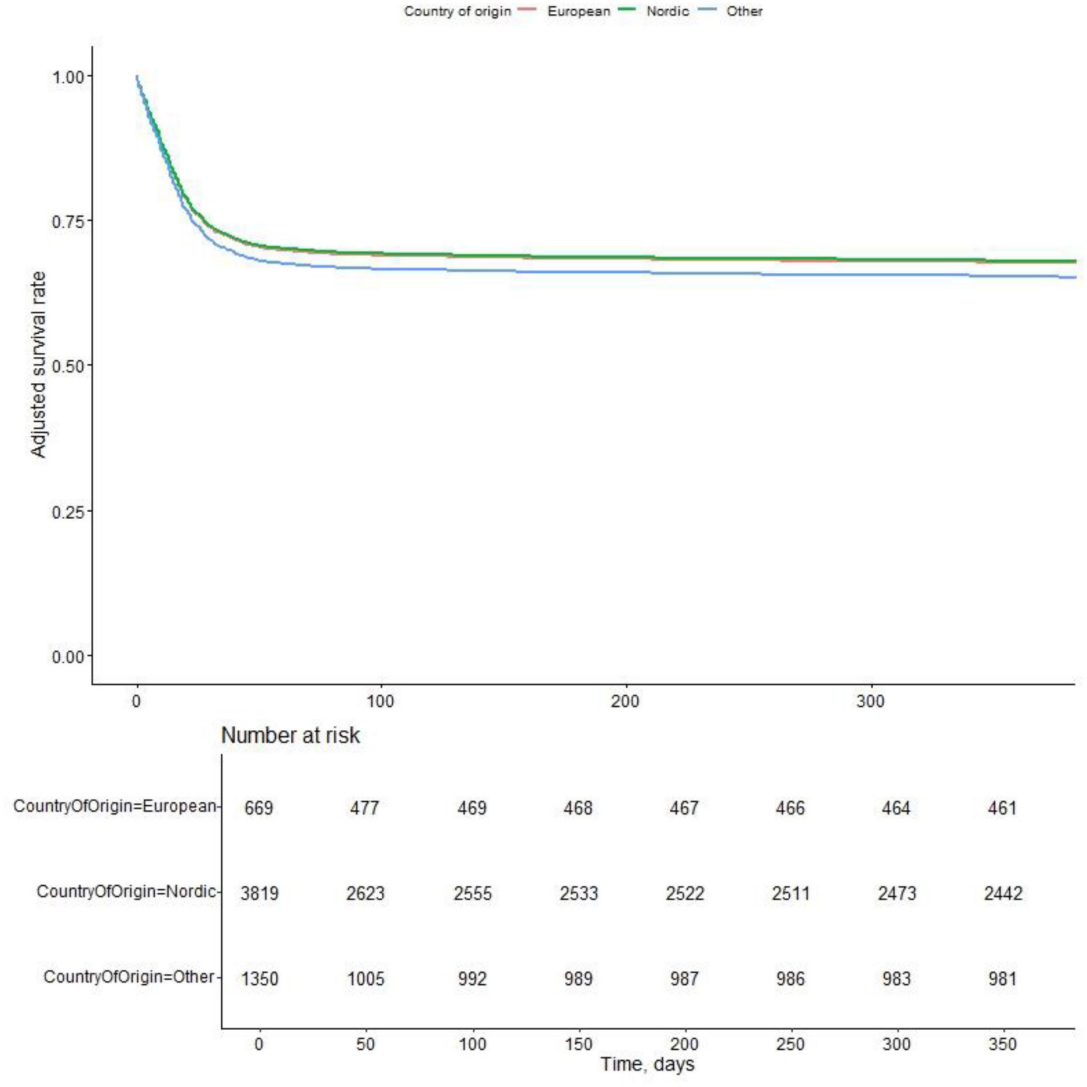
Adjusted Cox survival across country of origin. See online supplemental table 9 for Cox model. The Cox model assumptions of proportional hazards were evaluated using Schoenfeld residuals graphically and by testing for correlation between them and survival time. The assumption of proportional hazards was not met.

In the univariable analysis, mortality and organ-free support days varied across all country of origin and socioeconomic domains with the exception of marital status. In total, 5833 patients were included in the multivariable model and ‘other’ country of origin independently predicted mortality: adjusted OR (aOR) 1.34 [95% CI 1.13 to 1.61, p=0.001) 90 days after ICU admission. Furthermore, ‘other’ country of origin (ie, non-Nordic and non-European) was associated with mortality across all ancillary analyses—for patients under the age of 65 years; aOR 1.53 [1.18 to 1.98], p 0.001, for patients 65 years of age and older; aOR 1.34 [1.02 to 1.74], p=0.032 and patients in IMV aOR 1.50 [1.23 to 1.84], p<0.001 (online supplemental tables 2–4). There were inconsistent associations between the exposure variables and days free of organ support (online supplemental tables 5–7). None of the socioeconomic variables were associated with mortality, and there were significant interactions between both pandemic wave with income quartile and country of origin. Other independent predictors of 90-day mortality and organ support-free days were age, sex, chronic heart and lung disease, SAPS 3 score, immunosuppression, time in hospital prior to ICU admission, pandemic wave and admission PaO₂/FiO₂ ratio. The general estimating equation model allowing for index hospital clustering showed similar results as the main model (online supplemental table 8). An adjusted Cox survival plot across country of origin is found in figure 2 and online supplemental table 9. Baseline characteristic across country of origin is described in table 4.

**Table 4:** Baseline characteristics across country of origin

Variable	Overall	European	Nordic	Other	P	Missing (%)
No.	8025	952	5181	1889		
Mortality at day 90(%)	2415 (30.1)	259 (27.2)	1660 (32.0)	494 (26.2)	<0.001	0.0
Age (median (IQR))	64.0 (54.0, 72.0)	63.0 (54.0, 71.0)	66.0 (56.0, 74.0)	59.0 (49.0, 67.0)	<0.001	0.0
Age group (%)					<0.001	0.0
<50	1381 (17.2)	164 (17.2)	722 (13.9)	495 (26.2)		
50–59	1700 (21.2)	207 (21.7)	983 (19.0)	508 (26.9)		
60–69	2322 (28.9)	298 (31.3)	1468 (28.3)	555 (29.4)		
70–79	2130 (26.5)	229 (24.1)	1609 (31.1)	292 (15.5)		
80+	492 (6.1)	54 (5.7)	399 (7.7)	39 (2.1)		
Age≥65(%)	3800 (47.4)	428 (45.0)	2780 (53.7)	592 (31.3)	<0.001	0.0
Male sex (%)	5650 (70.4)	690 (72.5)	3609 (69.7)	1349 (71.4)	0.118	0.0
BMI (mean (SD))	30.6 (7.0)	31.7 (7.7)	30.6 (7.2)	29.8 (6.0)	<0.001	64.2
SAPS 3 score (median (IQR))	55.0 (49.0, 62.0)	54.0 (48.0, 61.0)	56.0 (50.0, 64.0)	53.0 (47.0, 59.0)	<0.001	0.0
Hypertension (%)	3620 (45.2)	453 (47.6)	2456 (47.6)	710 (37.6)	<0.001	0.2
Chronic heart disease (%)	1330 (16.6)	173 (18.2)	952 (18.4)	204 (10.8)	<0.001	0.2
Chronic lung disease (%)	1419 (17.7)	157 (16.5)	1005 (19.5)	257 (13.6)	<0.001	0.2
Severe obesity (%)	691 (8.6)	79 (8.3)	479 (9.3)	133 (7.0)	0.012	0.2
Diabetes mellitus (%)	2088 (26.1)	298 (31.3)	1271 (24.6)	518 (27.4)	<0.001	0.2
Chronic renal disease (%)	516 (6.4)	49 (5.1)	362 (7.0)	105 (5.6)	0.020	0.2
Chronic hepatic disease (%)	77 (1.0)	7 (0.7)	54 (1.0)	16 (0.8)	0.561	0.2
Chronic neuromuscular disease (%)	152 (1.9)	14 (1.5)	124 (2.4)	14 (0.7)	<0.001	0.2
Immunosuppression (%)	746 (9.3)	55 (5.8)	571 (11.1)	120 (6.4)	<0.001	0.2
COVID-19 vaccination (%)					<0.001	0.0
No	6471 (80.6)	811 (85.2)	4033 (77.8)	1625 (86.0)		
Partly	257 (3.2)	23 (2.4)	207 (4.0)	27 (1.4)		
Full	450 (5.6)	36 (3.8)	380 (7.3)	34 (1.8)		
Unknown	847 (10.6)	82 (8.6)	561 (10.8)	203 (10.7)		
PaO_2_/FiO_2_ (mean (SD))	14.2 (9.5)	13.4 (10.4)	14.3 (9.6)	14.1 (8.8)	0.084	24.1
Spent time in hospital prior to ICU admission (median (IQR))	2.0 (0.0, 4.0)	1.0 (0.0, 4.0)	2.0 (0.0, 4.0)	2.0 (0.0, 4.0)	0.044	0.0
Symptom onset prior to ICU admission (median (IQR))	10.5 (7.7, 13.5)	10.3 (7.6, 13.4)	10.5 (7.7, 13.6)	10.6 (7.9, 13.5)	0.109	0.2
Wave (%)					<0.001	0.0
Wave 1	2290 (28.5)	265 (27.8)	1306 (25.2)	718 (38.0)		
Wave 2	1943 (24.2)	265 (27.8)	1268 (24.5)	410 (21.7)		
Wave 3	2484 (31.0)	232 (24.4)	1731 (33.4)	519 (27.5)		
Wave 4	578 (7.2)	121 (12.7)	294 (5.7)	163 (8.6)		
Wave 5	730 (9.1)	69 (7.2)	582 (11.2)	79 (4.2)		
Income_quartiles (%)					<0.001	0.2
Q1	1946 (24.3)	325 (34.2)	766 (14.8)	852 (45.3)		
Q2	1907 (23.8)	222 (23.4)	1359 (26.3)	326 (17.3)		
Q3	2012 (25.1)	209 (22.0)	1409 (27.2)	394 (20.9)		
Q4	2145 (26.8)	194 (20.4)	1641 (31.7)	310 (16.5)		
Education_level (%)					<0.001	3.6
<9 years	1215 (15.7)	216 (24.2)	621 (12.2)	376 (21.6)		
>12 years	2017 (26.1)	212 (23.8)	1229 (24.1)	575 (33.1)		
10–12 years	3463 (44.7)	373 (41.9)	2509 (49.1)	581 (33.4)		
9 years	1045 (13.5)	90 (10.1)	749 (14.7)	206 (11.9)		
Household size>1 (%)	5886 (73.6)	740 (78.2)	3593 (69.6)	1550 (82.4)	<0.001	0.4
Registered partner (%)	4222 (56.5)	570 (64.3)	2451 (51.1)	1198 (67.0)	<0.001	6.8
Urban neighbourhood (%)	7234 (90.5)	921 (97.4)	4448 (86.2)	1862 (98.9)	<0.001	0.4

Numbers are No. (%) unless otherwise noted.

BMI, Body Mass Index.; ICU, intensive care unit; SAPS 3, Simplified Acute Physiology Score 3.

The E-value was 1.58 (1.25 - infinite) for the association between ‘other’ country of origin and 90-day mortality.

## Discussion

Among patients admitted to a Swedish ICU with COVID-19 and acute hypoxaemic respiratory failure, being born in a country outside Europe, but not other socioeconomic factors, was associated with an increased risk of death 90 days after ICU admission (aOR 1.34, 95% CI 1.13 to 1.61, p=0.001). This association was observed in both patients below and above 65 years old and also among the subgroup of patients treated with IMV. To the best of our knowledge, this is the first study to demonstrate the association in a nationwide ICU cohort in the Nordic countries and in Sweden where universal healthcare is a statutory right.

In the fully adjusted model, time spent in the hospital prior to ICU admission was strongly associated with increased mortality. This finding may be caused by a potential delay in receiving appropriate level of care, or it may be explained by different risk for patients needing intensive care early after symptoms compared with patients needing intensive care later.^9^ During the pandemic, many units faced capacity issues, which likely explains the delay in ICU admission. Given the link between country of origin and mortality, one could argue that delays to ICU-level admission could also be partly due to cultural and language barriers between patients, next of kin and healthcare staff.^10 11^ However, there was no significant correlation between country of origin and time from symptom onset to ICU admission in our dataset.

In contrast to the findings from Nordberg *et al*, our study found that income level had an uncertain association with both mortality and days free from organ support.^3^ This discrepancy may be due to population differences. Nordberg *et al* compared IMV-treated COVID-19 patients with matched non-COVID-19 controls, while we focused on the ICU population specifically.

Reassuringly, none of the socioeconomic factors in our model (income, education, marital status and population density) independently predicted 90-day mortality. Similarly, a recent paper analysing socioeconomic status in Swedish ICU patients prior to the pandemic found no consistent relationships between education, income and ICU death.^12^ Their analyses adjusted for being born outside of Sweden but not for country of origin.

However, our findings are in contrast with previous findings demonstrating the association between socioeconomic deprivation and mortality.^13 14^ There are several reasons for this divergence. First, universal health and a right to healthcare is mandated by law in Sweden, and the funding principles of healthcare differ between countries, which could potentially impact outcomes. Second, the methods used to describe socioeconomic status differ between the studies. Third, we only studied ICU patients, which makes our study cohort smaller. Additionally, we adjusted for several relevant confounders, including comorbidities, acute physiological derangement at the time of ICU admission and ICU treatment. Lastly, we included all pandemic waves in Sweden, in contrast to those reporting from the early phases of the pandemic only.

Our results align with those of several other studies. For example, a large, global meta-analysis found that certain ethnic groups had an increased risk of both ICU admission and death during the pandemic.^1^ Across 67 US hospitals, Hispanics admitted to an ICU had a 44% increased risk of death compared with Caucasians, even though they were younger and had a lower comorbidity burden.^15^

Overall, there is much to suggest that country of origin can affect outcomes after intensive care, but the big question is why. First, this association could be due to the risk for disease exposure and level of immunisation.^1 16^ In contrast to several other similar studies, we were able to adjust for vaccination status in our model, strengthening our conclusion that country of origin independently predicts mortality. Second, the link between genetic ancestry and mortality due to COVID-19 is not fully understood. Yet, recent research indicates that higher expression, polymorphisms, mutations and deletions of several genes are associated with the susceptibility, severity and clinical outcomes of COVID-19.^17^ Although country of origin is not synonymous with genetic ancestry, it may be a useful surrogate. Third, despite the legislative mandate for equitable healthcare in Sweden, systemic inequalities across different ethnic groups could impact ICU mortality during the pandemic, disproportionately affecting minorities. For example, mistrust of authorities^18^ and language barriers^10^ could lead to late presentation and challenges related to interpersonal communication. Lastly, disparities in the management of postintensive care syndrome patients exist, and it is unknown if this had an impact on the Swedish national ICU cohort.^19^

This study has several notable strengths. First, the data were prospectively collected through the well-established SIR at a national level, ensuring comprehensive and high-quality data. Ssecond, our analyses were meticulously adjusted for several relevant factors, enhancing the robustness and reliability of our findings. Third, the E-value of 1.58 is low compared with the OR for age but higher than the ORs for other confounders, indicating that the observed association is moderately robust in comparison with the potential of unmeasured confounding. This is further supported by the wide range of covariates included in the study—covering patient, ICU and socioeconomic variables—which reduces the likelihood of residual confounding. Regarding the association between ‘other’ country of origin and 90-day mortality, the E-values were similar for patients above and below 65 years of age. Lastly, the primary endpoint of the study had very few missing patients, which minimises bias and strengthens the validity of our conclusions.

Despite the strengths of our study, there are several limitations that should be acknowledged. First, we were unable to identify second-generation immigrants, which could potentially impact our results. This limitation may obscure the true effect of country-of-origin on ICU outcomes. Second, we did not use a deprivation index in our adjustments, which might have provided a more nuanced understanding of the socioeconomic factors at play. However, Sweden is known for its relatively low Gini index, reflecting a more equal distribution of income compared with many other countries. As of 2021, Sweden’s Gini index was 29.8, which is lower than the world average of 35.4, placing Sweden among the countries with the most equal income distribution.^20^ Third, while having included both education levels and neighbourhood metrics in our analysis, domains involving social support networks and cultural factors were not available to us and are unaccounted for in our model. It is entirely possible that disaggregated data may provide nuances for how different indicators of socioeconomic status may affect COVID-19 outcomes, in much the same way as disaggregated ethnicity data in the Open SAFELY dataset provided incremental information on COVID-19 outcomes in the UK.^21^ Fourth, we pooled non-Nordic and non-European patients into a single group of ‘other’ countries of origin due to the low numbers in individual countries in this category. This may mask important heterogeneity in marginalised groups. Fifth, we could not identify patients who received HFNO or intermittent haemodialysis outside of the ICU. This could lead to an underestimation of the severity of illness and the intensity of care required for some patients. Finally, we considered both exposures and covariates on an equal basis and cannot make any inferences about causality or effect modification.

## Conclusion

Despite Sweden’s universal healthcare system, this study found significant disparities in COVID-19 outcomes based on country of origin, however, not for socioeconomic factors. Patients born outside Europe had a higher risk of death 90 days after ICU admission. These findings suggest disparities in COVID-19 outcomes based on country of origin and could challenge the notion of equitable healthcare in Sweden. However, it remains possible that unmeasured socioeconomic indicators, patient and society-related factors—such as lifestyle, cultural differences and severity of pre-existing comorbidities—contribute to the observed differences in mortality. Further research using disaggregated data is needed to disentangle the effects of healthcare delivery from individual-level vulnerabilities and systemic factors.

## Supplementary material

10.1136/bmjopen-2025-099763 online supplemental file 1

## Data Availability

No data are available.
