# Supplementary material for: Impact of socioeconomic status and country of origin on COVID-19 outcomes in Swedish ICUs: a retrospective registry-based cohort study
Source: BMJ Open. 2025 Nov 16;15(11):e099763. doi: 10.1136/bmjopen-2025-099763 (PMC12625895; doi:10.1136/bmjopen-2025-099763)
Supplement: online supplemental file 1 [file bmjopen-15-11-s001.docx]

## Supplement 1. Logistic regression on 1-year mortality. 7926 patients included in the multivariable model. Global p-values for categorical variables are calculated using likelihood ratio tests.

| Covariate | Unadjusted OR(95%CI) | p | Adjusted OR(95%CI) | p (adj) |
| --- | --- | --- | --- | --- |
| Age group |  | <0.001 |  | <0.001 |
| <50 | Reference |  | Reference |  |
| 50-59 | 2.34 (1.88, 2.94) | <0.001 | 2.61 (1.98, 3.49) | <0.001 |
| 60-69 | 4.63 (3.77, 5.71) | <0.001 | 3.51 (2.68, 4.65) | <0.001 |
| 70-79 | 11.06 (9.04, 13.65) | <0.001 | 5.79 (4.34, 7.79) | <0.001 |
| 80+ | 21.05 (16.17, 27.58) | <0.001 | 10.05 (6.92, 14.73) | <0.001 |
| Sex |  | 0.008 |  | <0.001 |
| Female | Reference |  | Reference |  |
| Male | 1.15 (1.04, 1.28) | 0.008 | 1.32 (1.14, 1.53) | <0.001 |
| SAPS3 Score | 1.09 (1.09, 1.10) | <0.001 | 1.06 (1.05, 1.07) | <0.001 |
| Hypertension | 1.59 (1.44, 1.75) | <0.001 | 0.88 (0.77, 1.01) | 0.063 |
| Chronic heart disease | 2.69 (2.38, 3.03) | <0.001 | 1.34 (1.13, 1.57) | <0.001 |
| Chronic lung disease | 1.64 (1.46, 1.85) | <0.001 | 1.56 (1.32, 1.83) | <0.001 |
| Severe obesity | 0.69 (0.58, 0.83) | <0.001 | 1.14 (0.90, 1.45) | 0.27 |
| Diabetes mellitus | 1.41 (1.27, 1.57) | <0.001 | 1.12 (0.97, 1.30) | 0.13 |
| Chronic renal disease | 2.50 (2.08, 3.00) | <0.001 | 1.23 (0.96, 1.58) | 0.100 |
| Chronic hepatic disease | 2.75 (1.74, 4.37) | <0.001 | 0.99 (0.53, 1.87) | 0.98 |
| Chronic neuromuscular disease | 2.08 (1.48, 2.91) | <0.001 | 1.62 (0.99, 2.66) | 0.055 |
| Immunosuppression | 2.26 (1.94, 2.63) | <0.001 | 1.55 (1.26, 1.92) | <0.001 |
| Covid-19 vaccination |  | <0.001 |  | 0.37 |
| No | Reference |  | Reference |  |
| Partly | 1.85 (1.43, 2.39) | <0.001 | 1.21 (0.84, 1.72) | 0.30 |
| Full | 2.88 (2.35, 3.53) | <0.001 | 1.04 (0.74, 1.46) | 0.83 |
| Unknown | 1.27 (1.09, 1.48) | 0.002 | 1.21 (0.96, 1.51) | 0.11 |
| PaO2/FiO2 | 0.98 (0.97, 0.99) | <0.001 | 0.98 (0.97, 0.99) | <0.001 |
| Spent time in hospital prior to ICU admission | 1.07 (1.05, 1.08) | <0.001 | 1.03 (1.01, 1.04) | <0.001 |
| Symptom onset prior to ICU admission | 1.00 (0.99, 1.01) | 0.49 |  |  |
| Wave |  | <0.001 |  | 0.005 |
| Wave 1 | Reference |  | Reference |  |
| Wave 2 | 1.39 (1.22, 1.58) | <0.001 | 0.83 (0.69, 0.99) | 0.036 |
| Wave 3 | 1.00 (0.89, 1.14) | 0.95 | 0.77 (0.64, 0.92) | 0.005 |
| Wave 4 | 0.93 (0.76, 1.14) | 0.49 | 0.86 (0.63, 1.16) | 0.32 |
| Wave 5 | 3.03 (2.53, 3.63) | <0.001 | 1.15 (0.86, 1.55) | 0.34 |
| Income quartiles |  | <0.001 |  | 0.20 |
| Q4 | Reference |  | Reference |  |
| Q1 | 1.86 (1.62, 2.14) | <0.001 | 1.23 (1.00, 1.52) | 0.054 |
| Q2 | 2.55 (2.23, 2.93) | <0.001 | 1.21 (0.99, 1.47) | 0.059 |
| Q3 | 1.51 (1.31, 1.73) | <0.001 | 1.14 (0.95, 1.38) | 0.15 |
| Education level |  | <0.001 |  | 0.54 |
| >12 years | Reference |  | Reference |  |
| <9 years | 1.90 (1.64, 2.21) | <0.001 | 1.08 (0.88, 1.32) | 0.48 |
| 10-12 years | 1.05 (0.93, 1.18) | 0.46 | 0.98 (0.84, 1.15) | 0.82 |
| 9 years | 0.99 (0.84, 1.17) | 0.91 | 0.90 (0.72, 1.12) | 0.36 |
| Country-Of-Origin |  | <0.001 |  | 0.031 |
| Nordic | Reference |  | Reference |  |
| European | 0.74 (0.64, 0.86) | <0.001 | 1.02 (0.82, 1.25) | 0.88 |
| Other | 0.70 (0.62, 0.79) | <0.001 | 1.26 (1.06, 1.51) | 0.010 |
| Household size |  | <0.001 |  | 0.45 |
| Single-person household | Reference |  | Reference |  |
| Multi-person household | 0.67 (0.60, 0.74) | <0.001 | 0.95 (0.82, 1.09) | 0.45 |
| Marital status |  | 0.40 |  |  |
| No registered partner | Reference |  |  |  |
| Registered partner | 1.04 (0.94, 1.15) | 0.40 |  |  |
| Population density |  | 0.19 |  |  |
| Rural | Reference |  |  |  |
| Urban | 1.11 (0.95, 1.32) | 0.20 |  |  |

Supplement 2. Logistic regression on 90-day mortality < 65 years of age. 4225 patients, 3068 patients in multivariable model. The variables included in the multivariable model are the same as in the logistic regression on 90-day mortality in all age groups.

| Covariate | Unadjusted OR(95%CI) | p | Adjusted OR(95%CI) | p (adj) |
| --- | --- | --- | --- | --- |
| Age group |  | <0.001 |  | <0.001 |
| <50 | Reference |  | Reference |  |
| 50-59 | 2.32 (1.85, 2.93) | <0.001 | 2.59 (1.92, 3.48) | <0.001 |
| 60-69 | 3.40 (2.69, 4.32) | <0.001 | 2.55 (1.86, 3.50) | <0.001 |
| Sex |  | 0.044 |  | <0.001 |
| Female | Reference |  | Reference |  |
| Male | 1.21 (1.01, 1.46) |  | 1.52 (1.20, 1.94) |  |
| SAPS 3 Score | 1.09 (1.08, 1.10) | <0.001 | 1.07 (1.06, 1.09) | <0.001 |
| Hypertension | 1.30 (1.10, 1.55) | 0.002 | 0.91 (0.72, 1.15) | 0.43 |
| Chronic heart disease | 1.88 (1.43, 2.44) | <0.001 | 1.24 (0.87, 1.76) | 0.23 |
| Chronic lung disease | 1.69 (1.37, 2.07) | <0.001 | 1.60 (1.24, 2.07) | <0.001 |
| Severe obesity | 1.18 (0.92, 1.49) | 0.18 | 1.45 (1.07, 1.97) | 0.018 |
| Diabetes mellitus | 1.60 (1.33, 1.92) | <0.001 | 1.20 (0.94, 1.54) | 0.15 |
| Chronic renal disease | 1.78 (1.23, 2.53) | 0.002 | 1.03 (0.65, 1.66) | 0.89 |
| Chronic hepatic disease | 3.68 (1.95, 6.81) | <0.001 | 0.91 (0.39, 2.14) | 0.83 |
| Chronic neuromuscular disease | 1.89 (1.13, 3.04) | 0.012 | 1.39 (0.67, 2.87) | 0.37 |
| Immunosuppression | 2.17 (1.67, 2.79) | <0.001 | 1.33 (0.94, 1.88) | 0.11 |
| Covid-19 vaccination |  | <0.001 |  | 0.59 |
| No | Reference |  | Reference |  |
| Partly | 1.64 (0.94, 2.73) | 0.069 | 1.29 (0.63, 2.62) | 0.49 |
| Full | 2.41 (1.56, 3.65) | <0.001 | 1.23 (0.64, 2.35) | 0.53 |
| Unknown | 1.36 (1.06, 1.74) | 0.014 | 1.25 (0.88, 1.77) | 0.21 |
| PaO2/FiO2 | 0.98 (0.97, 0.99) | 0.005 | 0.98 (0.97, 1.00) | 0.023 |
| Spent time in hospital prior to ICU admission | 1.04 (1.02, 1.06) | <0.001 | 1.02 (1.00, 1.04) | 0.059 |
| Symptom onset prior to ICU admission | 1.01 (1.00, 1.03) | 0.16 |  |  |
| Wave |  | <0.001 |  | 0.082 |
| Wave 1 | Reference |  | Reference |  |
| Wave 2 | 0.90 (0.71, 1.14) | 0.38 | 0.69 (0.51, 0.93) | 0.016 |
| Wave 3 | 0.85 (0.69, 1.05) | 0.14 | 0.75 (0.56, 1.00) | 0.047 |
| Wave 4 | 0.80 (0.57, 1.11) | 0.19 | 0.85 (0.56, 1.31) | 0.47 |
| Wave 5 | 2.06 (1.50, 2.79) | <0.001 | 1.03 (0.62, 1.70) | 0.92 |
| Income quartiles |  | 0.002 |  | 0.49 |
| Q4 | Reference |  | Reference |  |
| Q1 | 1.35 (1.08, 1.68) | 0.008 | 1.13 (0.82, 1.54) | 0.46 |
| Q2 | 1.53 (1.19, 1.95) | <0.001 | 1.26 (0.91, 1.74) | 0.16 |
| Q3 | 1.08 (0.86, 1.35) | 0.50 | 1.18 (0.90, 1.55) | 0.23 |
| Education level |  | 0.31 |  | 0.90 |
| >12 years | Reference |  | Reference |  |
| <9 years | 1.34 (0.98, 1.82) | 0.067 | 1.03 (0.69, 1.54) | 0.87 |
| 10-12 years | 1.14 (0.93, 1.40) | 0.22 | 1.05 (0.82, 1.35) | 0.70 |
| 9 years | 1.13 (0.86, 1.49) | 0.37 | 0.93 (0.66, 1.32) | 0.70 |
| Country-Of-Origin |  | 0.49 |  | 0.004 |
| Nordic | Reference |  | Reference |  |
| European | 1.09 (0.84, 1.40) | 0.50 | 1.35 (0.97, 1.89) | 0.076 |
| Other | 1.11 (0.92, 1.33) | 0.26 | 1.53 (1.18, 1.98) | 0.001 |
| Household size |  | <0.001 |  | 0.46 |
| Single-person household | Reference |  | Reference |  |
| Multi-person household | 0.70 (0.58, 0.85) |  | 0.91 (0.71, 1.16) |  |
| Marital status |  | 0.51 |  |  |
| No registered partner | Reference |  |  |  |
| Registered partner | 0.95 (0.80, 1.12) |  |  |  |
| Population density |  | 0.19 |  |  |
| Rural | Reference |  |  |  |
| Urban | 1.24 (0.91, 1.73) |  |  |  |

## Supplement 3. Logistic regression on 90-day mortality > 65 years of age. 3800 patients, 2770 patients in multivariable model. The variables included in the multivariable model are the same as in the logistic regression on 90-day mortality in all age groups.

| Covariate | Unadjusted OR(95%CI) | p | Adjusted OR(95%CI) | p (adj) |
| --- | --- | --- | --- | --- |
| Age group |  | <0.001 |  | <0.001 |
| 60-69 | Reference |  | Reference |  |
| 70-79 | 1.76 (1.52, 2.04) | <0.001 | 1.36 (1.12, 1.65) | 0.002 |
| 80+ | 3.25 (2.62, 4.06) | <0.001 | 2.46 (1.82, 3.33) | <0.001 |
| Sex |  | 0.065 |  | 0.086 |
| Female | Reference |  | Reference |  |
| Male | 1.14 (0.99, 1.31) |  | 1.18 (0.98, 1.43) |  |
| SAPS 3 Score | 1.06 (1.05, 1.07) | <0.001 | 1.05 (1.04, 1.06) | <0.001 |
| Hypertension | 1.01 (0.88, 1.15) | 0.93 | 0.89 (0.75, 1.05) | 0.17 |
| Chronic heart disease | 1.61 (1.40, 1.87) | <0.001 | 1.29 (1.07, 1.55) | 0.007 |
| Chronic lung disease | 1.41 (1.20, 1.65) | <0.001 | 1.47 (1.19, 1.80) | <0.001 |
| Severe obesity | 0.78 (0.57, 1.07) | 0.13 | 0.85 (0.57, 1.25) | 0.40 |
| Diabetes mellitus | 1.01 (0.88, 1.16) | 0.93 | 1.03 (0.86, 1.24) | 0.74 |
| Chronic renal disease | 1.73 (1.38, 2.16) | <0.001 | 1.19 (0.89, 1.58) | 0.24 |
| Chronic hepatic disease | 1.58 (0.81, 3.14) | 0.18 | 0.71 (0.29, 1.70) | 0.44 |
| Chronic neuromuscular disease | 1.46 (0.90, 2.39) | 0.13 | 1.06 (0.56, 2.00) | 0.87 |
| Immunosuppression | 1.85 (1.50, 2.28) | <0.001 | 1.61 (1.23, 2.10) | <0.001 |
| Covid-19 vaccination |  | 0.37 |  | 0.83 |
| No | Reference |  | Reference |  |
| Partly | 1.06 (0.78, 1.43) | 0.72 | 1.07 (0.71, 1.61) | 0.74 |
| Full | 1.20 (0.96, 1.50) | 0.11 | 0.92 (0.62, 1.35) | 0.65 |
| Unknown | 1.11 (0.89, 1.37) | 0.35 | 1.10 (0.82, 1.48) | 0.53 |
| PaO2/FiO2 | 0.98 (0.97, 0.99) | <0.001 | 0.98 (0.97, 0.99) | <0.001 |
| Spent time in hospital prior to ICU admission | 1.05 (1.04, 1.07) | <0.001 | 1.03 (1.01, 1.04) | <0.001 |
| Symptom onset prior to ICU admission | 1.00 (0.99, 1.01) | 0.98 |  |  |
| Wave |  | 0.11 |  | 0.28 |
| Wave 1 | Reference |  | Reference |  |
| Wave 2 | 1.16 (0.97, 1.38) | 0.10 | 0.90 (0.71, 1.12) | 0.34 |
| Wave 3 | 0.96 (0.80, 1.14) | 0.61 | 0.82 (0.64, 1.05) | 0.12 |
| Wave 4 | 1.04 (0.77, 1.40) | 0.79 | 0.87 (0.57, 1.33) | 0.52 |
| Wave 5 | 1.19 (0.96, 1.49) | 0.12 | 0.68 (0.47, 0.97) | 0.034 |
| Income quartiles |  | <0.001 |  | 0.85 |
| Q4 | Reference |  | Reference |  |
| Q1 | 1.41 (1.15, 1.73) | <0.001 | 1.09 (0.81, 1.46) | 0.57 |
| Q2 | 1.59 (1.31, 1.93) | <0.001 | 1.12 (0.87, 1.45) | 0.38 |
| Q3 | 1.28 (1.04, 1.57) | 0.019 | 1.07 (0.83, 1.39) | 0.58 |
| Education levels |  | <0.001 |  | 0.41 |
| >12 years | Reference |  | Reference |  |
| <9 years | 1.25 (1.03, 1.51) | 0.023 | 1.04 (0.82, 1.34) | 0.73 |
| 10-12 years | 0.90 (0.76, 1.07) | 0.24 | 0.88 (0.72, 1.09) | 0.26 |
| 9 years | 0.83 (0.66, 1.04) | 0.10 | 0.90 (0.68, 1.21) | 0.49 |
| Country-Of-Origin |  | 0.051 |  | 0.076 |
| Nordic | Reference |  | Reference |  |
| European | 0.78 (0.63, 0.96) | 0.018 | 0.97 (0.74, 1.28) | 0.85 |
| Other | 1.02 (0.85, 1.21) | 0.85 | 1.34 (1.02, 1.74) | 0.032 |
| Household size |  | 0.050 |  | 0.81 |
| Single-person household | Reference |  | Reference |  |
| Multi-person household | 0.87 (0.76, 1.00) |  | 0.98 (0.82, 1.17) |  |
| Marital status |  | 0.12 |  |  |
| No registered partner | Reference |  |  |  |
| Registered partner | 0.89 (0.78, 1.03) |  |  |  |
| Population density |  | 0.008 |  |  |
| Rural | Reference |  |  |  |
| Urban | 1.33 (1.08, 1.64) |  |  |  |

## Supplement 4. Logistic regression on 90-day mortality, only IMV-treated. 5260 patients, 3931 patients in multivariable model.

| Covariate | Unadjusted OR(95%CI) | p | Adjusted OR(95%CI) | p (adj) |
| --- | --- | --- | --- | --- |
| Age group |  | <0.001 |  | <0.001 |
| <50 | Reference |  | Reference |  |
| 50-59 | 2.10 (1.65, 2.70) | <0.001 | 2.49 (1.84, 3.38) | <0.001 |
| 60-69 | 3.96 (3.17, 5.00) | <0.001 | 3.71 (2.76, 4.99) | <0.001 |
| 70-79 | 8.19 (6.54, 10.36) | <0.001 | 6.31 (4.58, 8.71) | <0.001 |
| 80+ | 16.10 (11.31, 23.16) | <0.001 | 12.63 (7.74, 20.61) | <0.001 |
| Sex |  | <0.001 |  | <0.001 |
| Female | Reference |  | Reference |  |
| Male | 1.25 (1.10, 1.42) |  | 1.35 (1.14, 1.60) |  |
| SAPS 3 Score | 1.07 (1.06, 1.08) | <0.001 | 1.04 (1.03, 1.05) | <0.001 |
| Hypertension | 1.44 (1.28, 1.61) | <0.001 | 0.88 (0.75, 1.03) | 0.100 |
| Chronic heart disease | 2.13 (1.83, 2.48) | <0.001 | 1.19 (0.97, 1.45) | 0.097 |
| Chronic lung disease | 1.48 (1.27, 1.71) | <0.001 | 1.48 (1.23, 1.79) | <0.001 |
| Severe obesity | 0.64 (0.52, 0.79) | <0.001 | 1.14 (0.87, 1.49) | 0.34 |
| Diabetes mellitus | 1.35 (1.19, 1.53) | <0.001 | 1.11 (0.93, 1.32) | 0.24 |
| Chronic renal disease | 1.89 (1.50, 2.38) | <0.001 | 1.08 (0.80, 1.46) | 0.63 |
| Chronic hepatic disease | 1.71 (0.99, 2.96) | 0.054 | 0.77 (0.37, 1.59) | 0.48 |
| Chronic neuromuscular disease | 1.16 (0.73, 1.80) | 0.52 |  |  |
| Immunosuppression | 2.03 (1.67, 2.46) | <0.001 | 1.71 (1.33, 2.21) | <0.001 |
| Covid-19 vaccination |  | <0.001 |  | 0.69 |
| No | Reference |  | Reference |  |
| Partly | 1.64 (1.18, 2.26) | 0.003 | 1.09 (0.71, 1.67) | 0.70 |
| Full | 2.14 (1.65, 2.77) | <0.001 | 0.90 (0.60, 1.36) | 0.63 |
| Unknown | 1.26 (1.05, 1.52) | 0.014 | 1.14 (0.88, 1.47) | 0.33 |
| PaO2/FiO2 | 0.98 (0.97, 0.99) | <0.001 | 0.98 (0.97, 0.99) | <0.001 |
| Spent time in hospital prior to ICU admission | 1.05 (1.04, 1.06) | <0.001 | 1.02 (1.01, 1.04) | <0.001 |
| Symptom onset prior to ICU admission | 1.01 (1.00, 1.02) | 0.035 | 0.99 (0.98, 1.00) | 0.13 |
| Wave |  | <0.001 |  | 0.90 |
| Wave 1 | Reference |  | Reference |  |
| Wave 2 | 1.62 (1.39, 1.89) | <0.001 | 1.05 (0.86, 1.29) | 0.62 |
| Wave 3 | 1.27 (1.10, 1.47) | 0.001 | 1.02 (0.83, 1.26) | 0.83 |
| Wave 4 | 1.29 (1.00, 1.64) | 0.044 | 1.18 (0.83, 1.67) | 0.36 |
| Wave 5 | 2.07 (1.65, 2.59) | <0.001 | 1.01 (0.71, 1.43) | 0.97 |
| Income quartiles |  | <0.001 |  | 0.75 |
| Q4 | Reference |  | Reference |  |
| Q1 | 1.41 (1.20, 1.66) | <0.001 | 0.95 (0.75, 1.21) | 0.68 |
| Q2 | 1.89 (1.61, 2.22) | <0.001 | 1.03 (0.82, 1.28) | 0.82 |
| Q3 | 1.29 (1.10, 1.52) | 0.002 | 1.07 (0.87, 1.32) | 0.51 |
| Education levels |  | <0.001 |  | 0.65 |
| >12 years | Reference |  | Reference |  |
| <9 years | 1.60 (1.33, 1.92) | <0.001 | 1.07 (0.84, 1.35) | 0.60 |
| 10-12 years | 0.98 (0.85, 1.13) | 0.78 | 0.98 (0.82, 1.17) | 0.81 |
| 9 years | 0.92 (0.75, 1.12) | 0.40 | 0.89 (0.69, 1.15) | 0.37 |
| Country-Of-Origin |  | 0.066 |  | <0.001 |
| Nordic | Reference |  | Reference |  |
| European | 0.84 (0.69, 1.00) | 0.056 | 1.14 (0.89, 1.46) | 0.29 |
| Other | 0.89 (0.78, 1.02) | 0.093 | 1.50 (1.23, 1.84) | <0.001 |
| Household size |  | <0.001 |  | 0.80 |
| Single-person household | Reference |  | Reference |  |
| Multi-person household | 0.78 (0.68, 0.88) |  | 0.98 (0.83, 1.16) |  |
| Marital status |  | 0.16 |  |  |
| No registered partner | Reference |  |  |  |
| Registered partner | 1.09 (0.97, 1.23) |  |  |  |
| Population density |  | 0.092 |  | 0.036 |
| Rural | Reference |  | Reference |  |
| Urban | 1.19 (0.97, 1.45) |  | 1.33 (1.02, 1.72) |  |

## Supplement 5. Ordinal regression on Ventilator and CRRT free days within 60 days. 5539 patients were included in the multivariable model. 542 patients missed the outcome.

| Covariate | Unadjusted OR(95%CI) | p | Adjusted OR(95%CI) | p (adj) |
| --- | --- | --- | --- | --- |
| Age group |  |  |  | <0.001 |
| <50 | Reference |  | Reference |  |
| 50-59 | 0.79 (0.70, 0.91) | <0.001 | 0.85 (0.72, 0.99) | 0.034 |
| 60-69 | 0.56 (0.49, 0.63) | <0.001 | 0.83 (0.71, 0.98) | 0.023 |
| 70-79 | 0.28 (0.24, 0.32) | <0.001 | 0.61 (0.51, 0.74) | <0.001 |
| 80+ | 0.16 (0.13, 0.20) | <0.001 | 0.40 (0.30, 0.55) | <0.001 |
| Sex |  |  |  | 0.005 |
| Female | Reference |  | Reference |  |
| Male | 0.92 (0.84, 1.01) | 0.069 | 0.86 (0.77, 0.96) | 0.006 |
| SAPS3 Score | 0.93 (0.93, 0.94) | <0.001 | 0.94 (0.94, 0.95) | <0.001 |
| Hypertension | 0.75 (0.69, 0.82) | <0.001 | 1.05 (0.94, 1.17) | 0.37 |
| Chronic heart disease | 0.52 (0.46, 0.58) | <0.001 | 0.88 (0.76, 1.01) | 0.078 |
| Chronic lung disease | 0.73 (0.66, 0.81) | <0.001 | 0.81 (0.71, 0.93) | 0.002 |
| Severe obesity | 1.14 (1.00, 1.31) | 0.054 | 0.85 (0.72, 1.01) | 0.064 |
| Diabetes mellitus | 0.83 (0.75, 0.91) | <0.001 | 1.02 (0.91, 1.15) | 0.75 |
| Chronic renal disease | 0.56 (0.47, 0.66) | <0.001 | 0.99 (0.80, 1.22) | 0.89 |
| Chronic hepatic disease | 0.56 (0.37, 0.85) | 0.007 | 1.21 (0.73, 2.01) | 0.45 |
| Chronic neuromuscular disease | 0.87 (0.64, 1.18) | 0.36 | 1.16 (0.78, 1.72) | 0.47 |
| Immunosuppression | 0.62 (0.54, 0.72) | <0.001 | 0.91 (0.76, 1.09) | 0.29 |
| Covid-19 vaccination |  |  |  | 0.52 |
| No | Reference |  | Reference |  |
| Partly | 0.75 (0.60, 0.95) | 0.016 | 0.92 (0.69, 1.23) | 0.58 |
| Full | 0.62 (0.51, 0.74) | <0.001 | 1.05 (0.80, 1.39) | 0.73 |
| Unknown | 0.82 (0.71, 0.94) | 0.005 | 0.85 (0.71, 1.02) | 0.076 |
| PaO2FiO2 | 1.01 (1.01, 1.02) | <0.001 | 1.01 (1.00, 1.01) | <0.001 |
| Spent time in hospital prior to ICU admission | 0.96 (0.95, 0.97) | <0.001 | 0.99 (0.98, 1.00) | 0.090 |
| Symptom onset prior to ICU admission | 0.99 (0.99, 1.00) | 0.091 | 1.00 (0.99, 1.01) | 0.95 |
| Wave |  |  |  | <0.001 |
| Wave 1 | Reference |  | Reference |  |
| Wave 2 | 0.94 (0.84, 1.06) | 0.31 | 1.32 (1.15, 1.52) | <0.001 |
| Wave 3 | 1.12 (1.01, 1.24) | 0.034 | 1.29 (1.12, 1.48) | <0.001 |
| Wave 4 | 1.35 (1.14, 1.60) | <0.001 | 1.37 (1.10, 1.71) | 0.004 |
| Wave 5 | 0.71 (0.61, 0.83) | <0.001 | 1.39 (1.10, 1.75) | 0.006 |
| Income quartiles |  |  |  | <0.001 |
| Q4 | Reference |  | Reference |  |
| Q1 | 0.62 (0.55, 0.69) | <0.001 | 0.74 (0.63, 0.86) | <0.001 |
| Q2 | 0.51 (0.45, 0.57) | <0.001 | 0.75 (0.65, 0.87) | <0.001 |
| Q3 | 0.76 (0.68, 0.85) | <0.001 | 0.81 (0.71, 0.93) | 0.003 |
| Education level |  |  |  | 0.89 |
| >12 years | Reference |  | Reference |  |
| <9 years | 0.61 (0.53, 0.70) | <0.001 | 0.97 (0.82, 1.15) | 0.71 |
| 10-12 years | 0.98 (0.88, 1.08) | 0.68 | 1.06 (0.94, 1.20) | 0.34 |
| 9 years | 0.98 (0.85, 1.12) | 0.73 | 1.07 (0.91, 1.26) | 0.42 |
| Country-Of-Origin |  |  |  | 0.25 |
| Nordic | Reference |  | Reference |  |
| European | 1.11 (0.98, 1.27) | 0.11 | 0.97 (0.82, 1.14) | 0.69 |
| Other | 1.12 (1.02, 1.23) | 0.022 | 0.87 (0.77, 1.00) | 0.049 |
| Household size |  |  |  | 0.88 |
| Single-person household | Reference |  | Reference |  |
| Multi-person household | 1.29 (1.18, 1.42) | <0.001 | 0.99 (0.88, 1.11) | 0.89 |
| Marital status |  |  |  |  |
| No registered partner | Reference |  |  |  |
| Registered partner | 0.93 (0.86, 1.02) | 0.12 |  |  |
| Population density |  |  |  | 0.43 |
| Rural | Reference |  | Reference |  |
| Urban | 0.86 (0.75, 0.98) | 0.028 | 0.89 (0.75, 1.06) | 0.18 |

## Supplement 6. Ordinal regression on Ventilator free days within 60 days. 5838 patients included in the multivariable model.

| Covariate | Unadjusted Estimate(95%CI) | p | Adjusted OR(95%CI) | p (adj) |
| --- | --- | --- | --- | --- |
| Age group |  | <0.001 |  | <0.001 |
| <50 | Reference |  | Reference |  |
| 50-59 | -2.47 (-4.22, -0.73) | 0.006 | 0.88 (0.75, 1.02) | 0.081 |
| 60-69 | -5.89 (-7.53, -4.25) | <0.001 | 0.87 (0.75, 1.01) | 0.077 |
| 70-79 | -11.81 (-13.48, -10.14) | <0.001 | 0.67 (0.56, 0.80) | <0.001 |
| 80+ | -13.77 (-16.30, -11.23) | <0.001 | 0.44 (0.33, 0.59) | <0.001 |
| Sex |  | 0.46 |  |  |
| Female | Reference |  |  |  |
| Male | -0.45 (-1.65, 0.75) | 0.46 |  |  |
| SAPS3 Score | -0.67 (-0.72, -0.63) | <0.001 | 0.94 (0.94, 0.95) | <0.001 |
| Hypertension | -2.68 (-3.78, -1.58) | <0.001 | 1.04 (0.94, 1.16) | 0.41 |
| Chronic heart disease | -5.03 (-6.50, -3.56) | <0.001 | 0.86 (0.75, 0.99) | 0.035 |
| Chronic lung disease | -2.33 (-3.76, -0.89) | 0.002 | 0.82 (0.72, 0.93) | 0.002 |
| Severe obesity | -0.20 (-2.16, 1.75) | 0.84 |  |  |
| Diabetes mellitus | -1.77 (-3.02, -0.52) | 0.005 | 1.00 (0.89, 1.12) | 0.99 |
| Chronic renal disease | -4.23 (-6.46, -2.00) | <0.001 | 1.01 (0.82, 1.25) | 0.89 |
| Chronic hepatic disease | -7.58 (-13.20, -1.96) | 0.008 | 1.13 (0.69, 1.84) | 0.64 |
| Chronic neuromuscular disease | -0.37 (-4.39, 3.65) | 0.86 |  |  |
| Immunosuppression | -3.59 (-5.47, -1.70) | <0.001 | 0.92 (0.78, 1.10) | 0.38 |
| Covid-19 vaccination |  | 0.077 |  | 0.52 |
| No | Reference |  | Reference |  |
| Partly | -2.43 (-5.55, 0.69) | 0.13 | 0.89 (0.66, 1.19) | 0.43 |
| Full | -2.69 (-5.08, -0.29) | 0.028 | 1.08 (0.82, 1.42) | 0.58 |
| Unknown | -0.19 (-1.98, 1.61) | 0.84 | 0.90 (0.76, 1.06) | 0.22 |
| PaO2FiO2 | 0.19 (0.12, 0.25) | <0.001 | 1.01 (1.00, 1.01) | <0.001 |
| Spent time in hospital prior to ICU admission | -0.34 (-0.44, -0.23) | <0.001 | 0.99 (0.98, 1.00) | 0.10 |
| Symptom onset prior to ICU admission | -0.05 (-0.15, 0.04) | 0.24 |  |  |
| Wave |  | <0.001 |  | <0.001 |
| Wave 1 | Reference |  | Reference |  |
| Wave 2 | 2.49 (0.98, 4.00) | 0.001 | 1.31 (1.15, 1.50) | <0.001 |
| Wave 3 | 3.49 (2.07, 4.90) | <0.001 | 1.27 (1.11, 1.45) | <0.001 |
| Wave 4 | 6.31 (4.03, 8.59) | <0.001 | 1.35 (1.09, 1.67) | 0.006 |
| Wave 5 | 0.14 (-1.94, 2.22) | 0.89 | 1.34 (1.06, 1.68) | 0.013 |
| Income quartiles |  | <0.001 |  | <0.001 |
| Q4 | Reference |  | Reference |  |
| Q1 | -6.10 (-7.63, -4.57) | <0.001 | 0.77 (0.67, 0.90) | <0.001 |
| Q2 | -7.31 (-8.85, -5.78) | <0.001 | 0.78 (0.68, 0.90) | <0.001 |
| Q3 | -3.54 (-5.06, -2.03) | <0.001 | 0.82 (0.72, 0.93) | 0.002 |
| Education level |  | <0.001 |  | 0.56 |
| >12 years | Reference |  | Reference |  |
| <9 years | -5.29 (-7.07, -3.51) | <0.001 | 0.95 (0.81, 1.12) | 0.57 |
| 10-12 years | -0.59 (-1.96, 0.79) | 0.40 | 1.05 (0.93, 1.18) | 0.42 |
| 9 years | -0.44 (-2.31, 1.43) | 0.65 | 1.07 (0.91, 1.26) | 0.41 |
| Country-Of-Origin |  | 0.055 |  | 0.067 |
| Nordic | Reference |  | Reference |  |
| European | 1.96 (0.23, 3.69) | 0.027 | 1.01 (0.86, 1.18) | 0.94 |
| Other | 0.93 (-0.39, 2.25) | 0.17 | 0.86 (0.76, 0.98) | 0.024 |
| Household size |  | <0.001 |  | 0.57 |
| Single-person household | Reference |  | Reference |  |
| Multi-person household | 2.30 (1.05, 3.54) | <0.001 | 0.98 (0.88, 1.10) | 0.77 |
| Marital status |  | 0.18 |  |  |
| No registered partner | Reference |  |  |  |
| Registered partner | -0.79 (-1.93, 0.36) | 0.18 |  |  |
| Population density |  | 0.34 |  |  |
| Rural | Reference |  |  |  |
| Urban | -0.90 (-2.78, 0.97) | 0.34 |  |  |

## Supplement 7. Ordinal regression on CRRT free days within 60 days. 5539 patients were included in the multivariable model. 542 patients missed the outcome.

| Covariate | Unadjusted Estimate(95%CI) | p | Adjusted OR(95%CI) | p (adj) |
| --- | --- | --- | --- | --- |
| Age group |  | <0.001 |  | <0.001 |
| <50 | Reference |  | Reference |  |
| 50-59 | -5.72 (-7.68, -3.77) | <0.001 | 0.50 (0.39, 0.63) | <0.001 |
| 60-69 | -12.83 (-14.66, -10.99) | <0.001 | 0.41 (0.33, 0.52) | <0.001 |
| 70-79 | -23.17 (-25.03, -21.32) | <0.001 | 0.27 (0.21, 0.35) | <0.001 |
| 80+ | -31.41 (-34.19, -28.62) | <0.001 | 0.15 (0.11, 0.22) | <0.001 |
| Sex |  | <0.001 |  | <0.001 |
| Female | Reference |  | Reference |  |
| Male | -2.80 (-4.18, -1.41) | <0.001 | 0.76 (0.66, 0.87) | <0.001 |
| SAPS3 Score | -0.96 (-1.02, -0.91) | <0.001 | 0.94 (0.94, 0.95) | <0.001 |
| Hypertension | -6.34 (-7.61, -5.08) | <0.001 | 1.06 (0.93, 1.20) | 0.40 |
| Chronic heart disease | -12.38 (-14.05, -10.71) | <0.001 | 0.78 (0.67, 0.92) | 0.003 |
| Chronic lung disease | -5.62 (-7.27, -3.97) | <0.001 | 0.72 (0.62, 0.84) | <0.001 |
| Severe obesity | 3.58 (1.33, 5.82) | 0.002 | 0.83 (0.66, 1.03) | 0.087 |
| Diabetes mellitus | -4.62 (-6.07, -3.18) | <0.001 | 0.94 (0.82, 1.08) | 0.41 |
| Chronic renal disease | -16.68 (-19.18, -14.19) | <0.001 | 0.65 (0.52, 0.81) | <0.001 |
| Chronic hepatic disease | -8.59 (-14.92, -2.27) | 0.008 | 1.27 (0.69, 2.33) | 0.44 |
| Chronic neuromuscular disease | -3.83 (-8.42, 0.76) | 0.10 | 0.89 (0.56, 1.41) | 0.62 |
| Immunosuppression | -9.23 (-11.38, -7.09) | <0.001 | 0.74 (0.60, 0.90) | 0.003 |
| Covid-19 vaccination |  | <0.001 |  | 0.30 |
| No | Reference |  | Reference |  |
| Partly | -3.46 (-6.95, 0.02) | 0.051 | 0.95 (0.67, 1.34) | 0.77 |
| Full | -9.45 (-12.16, -6.73) | <0.001 | 0.95 (0.69, 1.30) | 0.73 |
| Unknown | -2.67 (-4.79, -0.54) | 0.014 | 0.79 (0.63, 0.98) | 0.032 |
| PaO2FiO2 | 0.24 (0.17, 0.32) | <0.001 | 1.02 (1.01, 1.03) | <0.001 |
| Spent time in hospital prior to ICU admission | -0.63 (-0.75, -0.51) | <0.001 | 0.98 (0.97, 0.99) | <0.001 |
| Symptom onset prior to ICU admission | -0.08 (-0.18, 0.03) | 0.14 |  |  |
| Wave |  | <0.001 |  | <0.001 |
| Wave 1 | Reference |  | Reference |  |
| Wave 2 | -1.10 (-2.86, 0.65) | 0.22 | 1.55 (1.31, 1.83) | <0.001 |
| Wave 3 | 3.06 (1.41, 4.70) | <0.001 | 1.63 (1.37, 1.94) | <0.001 |
| Wave 4 | 4.54 (1.91, 7.17) | <0.001 | 1.53 (1.15, 2.03) | 0.003 |
| Wave 5 | -5.73 (-8.10, -3.36) | <0.001 | 1.85 (1.40, 2.44) | <0.001 |
| Income quartiles |  | <0.001 |  | 0.10 |
| Q4 | Reference |  | Reference |  |
| Q1 | -6.68 (-8.45, -4.91) | <0.001 | 0.84 (0.69, 1.03) | 0.088 |
| Q2 | -10.71 (-12.47, -8.94) | <0.001 | 0.84 (0.70, 1.00) | 0.056 |
| Q3 | -4.31 (-6.06, -2.56) | <0.001 | 0.88 (0.74, 1.05) | 0.15 |
| Education level |  | <0.001 |  | 0.86 |
| >12 years | Reference |  | Reference |  |
| <9 years | -8.35 (-10.40, -6.30) | <0.001 | 0.95 (0.78, 1.15) | 0.59 |
| 10-12 years | -0.05 (-1.63, 1.53) | 0.95 | 1.06 (0.91, 1.24) | 0.43 |
| 9 years | 0.15 (-2.00, 2.30) | 0.89 | 1.05 (0.86, 1.29) | 0.62 |
| Country-Of-Origin |  | 0.001 |  | 0.061 |
| Nordic | Reference |  | Reference |  |
| European | 1.65 (-0.41, 3.71) | 0.12 | 0.91 (0.74, 1.11) | 0.36 |
| Other | 2.77 (1.24, 4.30) | <0.001 | 0.79 (0.67, 0.93) | 0.006 |
| Household size |  | <0.001 |  | 0.11 |
| Single-person household | Reference |  | Reference |  |
| Multi-person household | 4.92 (3.49, 6.36) | <0.001 | 1.05 (0.91, 1.20) | 0.49 |
| Marital status |  | 0.15 |  |  |
| No registered partner | Reference |  |  |  |
| Registered partner | -0.96 (-2.28, 0.36) | 0.15 |  |  |
| Population density |  | 0.021 |  | 0.14 |
| Rural | Reference |  | Reference |  |
| Urban | -2.49 (-4.60, -0.38) | 0.021 | 0.90 (0.73, 1.10) | 0.31 |

## Supplement 8. Generalised estimating equations logistic regression on 90-day mortality, clustered for hospital. 5459 patients in the multivariable model. Fever than in the other models as marital status was included in the model.

| Covariate | Unadjusted OR(95%CI) | p | Adjusted OR(95%CI) | p (adj) |
| --- | --- | --- | --- | --- |
| Age group |  | <0.001 |  | <0.001 |
| <50 | Reference |  | Reference |  |
| 50-59 | 2.69 (2.08, 3.48) | <0.001 | 2.50 (1.92, 3.25) | <0.001 |
| 60-69 | 5.01 (3.81, 6.60) | <0.001 | 3.44 (2.60, 4.54) | <0.001 |
| 70-79 | 11.69 (8.51, 16.05) | <0.001 | 5.69 (4.08, 7.94) | <0.001 |
| 80+ | 25.02 (16.77, 37.32) | <0.001 | 10.40 (6.68, 16.20) | <0.001 |
| Sex |  | 0.018 |  | 0.012 |
| Female | Reference |  | Reference |  |
| Male | 1.20 (1.03, 1.40) | 0.018 | 1.29 (1.06, 1.57) | 0.012 |
| SAPS3 Score | 1.09 (1.08, 1.10) | <0.001 | 1.06 (1.05, 1.07) | <0.001 |
| Hypertension | 1.56 (1.34, 1.82) | <0.001 | 0.90 (0.79, 1.03) | 0.13 |
| Chronic heart disease | 2.45 (2.13, 2.82) | <0.001 | 1.28 (1.15, 1.43) | <0.001 |
| Chronic lung disease | 1.52 (1.28, 1.80) | <0.001 | 1.43 (1.24, 1.66) | <0.001 |
| Severe obesity | 0.72 (0.56, 0.92) | 0.009 | 1.19 (0.90, 1.57) | 0.22 |
| Diabetes mellitus | 1.37 (1.17, 1.61) | <0.001 | 1.09 (0.92, 1.30) | 0.33 |
| Chronic renal disease | 2.30 (1.92, 2.77) | <0.001 | 1.13 (0.93, 1.38) | 0.20 |
| Chronic hepatic disease | 1.97 (1.00, 3.87) | 0.049 | 1.03 (0.48, 2.23) | 0.94 |
| Chronic neuromuscular disease | 1.27 (0.84, 1.92) | 0.26 |  |  |
| Immunosuppression | 2.01 (1.68, 2.40) | <0.001 | 1.57 (1.26, 1.96) | <0.001 |
| Covid-19 vaccination |  | <0.001 |  | 0.68 |
| No | Reference |  | Reference |  |
| Partly | 1.56 (1.18, 2.06) | 0.002 | 1.07 (0.75, 1.51) | 0.71 |
| Full | 2.09 (1.65, 2.64) | <0.001 | 0.92 (0.68, 1.24) | 0.58 |
| Unknown | 1.14 (0.88, 1.48) | 0.32 | 1.14 (0.89, 1.45) | 0.29 |
| PaO2FiO2 | 0.97 (0.96, 0.99) | <0.001 | 0.98 (0.97, 0.99) | <0.001 |
| Spent time in hospital prior to ICU admission | 1.06 (1.04, 1.08) | <0.001 | 1.02 (1.01, 1.04) | <0.001 |
| Symptom onset prior to ICU admission | 1.01 (0.99, 1.02) | 0.40 |  |  |
| Wave |  | <0.001 |  | 0.10 |
| Wave 1 | Reference |  | Reference |  |
| Wave 2 | 1.31 (1.01, 1.70) | 0.044 | 0.80 (0.65, 0.99) | 0.042 |
| Wave 3 | 1.00 (0.82, 1.22) | 0.97 | 0.79 (0.64, 0.97) | 0.026 |
| Wave 4 | 0.95 (0.74, 1.22) | 0.71 | 0.84 (0.63, 1.10) | 0.21 |
| Wave 5 | 1.86 (1.40, 2.48) | <0.001 | 0.76 (0.56, 1.02) | 0.068 |
| Income quartiles |  | <0.001 |  | 0.029 |
| Q4 | Reference |  | Reference |  |
| Q1 | 1.65 (1.37, 1.99) | <0.001 | 1.16 (0.95, 1.41) | 0.14 |
| Q2 | 2.31 (1.91, 2.79) | <0.001 | 1.21 (1.06, 1.39) | 0.006 |
| Q3 | 1.53 (1.29, 1.80) | <0.001 | 1.20 (1.02, 1.43) | 0.031 |
| Education level |  | <0.001 |  | 0.28 |
| >12 years | Reference |  | Reference |  |
| <9 years | 1.74 (1.43, 2.12) | <0.001 | 1.10 (0.91, 1.31) | 0.32 |
| 10-12 years | 1.01 (0.85, 1.19) | 0.94 | 0.98 (0.83, 1.15) | 0.78 |
| 9 years | 0.91 (0.74, 1.12) | 0.39 | 0.90 (0.73, 1.11) | 0.33 |
| Country-Of-Origin |  | 0.011 |  | <0.001 |
| Nordic | Reference |  | Reference |  |
| European | 0.90 (0.72, 1.12) | 0.33 | 1.12 (0.91, 1.38) | 0.29 |
| Other | 0.78 (0.66, 0.92) | 0.003 | 1.38 (1.17, 1.63) | <0.001 |
| Household size |  | <0.001 |  | 0.14 |
| Single-person household | Reference |  | Reference |  |
| Multi-person household | 0.79 (0.68, 0.90) | <0.001 | 0.89 (0.77, 1.04) | 0.14 |
| Marital status |  | 0.017 |  | 0.71 |
| No registered partner | Reference |  | Reference |  |
| Registered partner | 1.15 (1.03, 1.29) | 0.017 | 1.04 (0.86, 1.25) | 0.71 |
| Population density |  | 0.47 |  |  |
| Rural | Reference |  |  |  |
| Urban | 1.09 (0.86, 1.37) | 0.47 |  |  |

## Supplement 9. Cox regression on mortality. 5452 patients having complete data. After excluding variables with p>0.1 in the univariable analysis, 5838 patients were included in the multivariable model. The assumption of proportional hazards was not met according to the Shoenfield residuals method.

| Covariate | Unadjusted HR(95%CI) | p | Adjusted HR(95%CI) | p (adj) |
| --- | --- | --- | --- | --- |
| Age group |  |  |  |  |
| <50 | Reference |  | Reference |  |
| 50-59 | 2.22 (1.81, 2.73) | <0.001 | 2.36 (1.84, 3.03) | <0.001 |
| 60-69 | 4.03 (3.33, 4.87) | <0.001 | 3.03 (2.39, 3.84) | <0.001 |
| 70-79 | 7.96 (6.62, 9.58) | <0.001 | 4.35 (3.41, 5.55) | <0.001 |
| 80+ | 13.18 (10.72, 16.19) | <0.001 | 6.65 (5.04, 8.78) | <0.001 |
| Sex |  |  |  |  |
| Female | Reference |  | Reference |  |
| Male | 1.08 (0.99, 1.17) | 0.078 | 1.13 (1.02, 1.25) | 0.023 |
| SAPS3 Score | 1.06 (1.06, 1.07) | <0.001 | 1.05 (1.04, 1.05) | <0.001 |
| Hypertension | 1.48 (1.37, 1.59) | <0.001 | 0.90 (0.82, 0.99) | 0.028 |
| Chronic heart disease | 2.21 (2.03, 2.41) | <0.001 | 1.27 (1.14, 1.41) | <0.001 |
| Chronic lung disease | 1.50 (1.37, 1.64) | <0.001 | 1.39 (1.24, 1.55) | <0.001 |
| Severe obesity | 0.74 (0.63, 0.86) | <0.001 | 1.07 (0.90, 1.28) | 0.42 |
| Diabetes mellitus | 1.34 (1.24, 1.46) | <0.001 | 1.11 (1.01, 1.23) | 0.039 |
| Chronic renal disease | 2.13 (1.89, 2.41) | <0.001 | 1.17 (1.01, 1.36) | 0.039 |
| Chronic hepatic disease | 2.08 (1.54, 2.80) | <0.001 | 0.93 (0.64, 1.36) | 0.73 |
| Chronic neuromuscular disease | 1.54 (1.21, 1.96) | <0.001 | 1.30 (0.96, 1.76) | 0.093 |
| Immunosuppression | 1.93 (1.73, 2.15) | <0.001 | 1.43 (1.26, 1.64) | <0.001 |
| Covid-19 vaccination |  |  |  |  |
| No | Reference |  | Reference |  |
| Partly | 1.57 (1.30, 1.89) | <0.001 | 1.14 (0.90, 1.45) | 0.27 |
| Full | 2.00 (1.75, 2.29) | <0.001 | 1.08 (0.87, 1.34) | 0.47 |
| Unknown | 1.17 (1.04, 1.33) | 0.010 | 1.16 (0.99, 1.36) | 0.070 |
| PaO2FiO2 | 0.98 (0.98, 0.99) | <0.001 | 0.99 (0.98, 0.99) | <0.001 |
| Spent time in hospital prior to ICU admission | 1.03 (1.02, 1.03) | <0.001 | 1.01 (1.00, 1.01) | 0.007 |
| Symptom onset prior to ICU admission | 1.00 (1.00, 1.01) | 0.30 |  |  |
| Wave |  |  |  |  |
| Wave 1 | Reference |  | Reference |  |
| Wave 2 | 1.30 (1.17, 1.44) | <0.001 | 0.86 (0.76, 0.97) | 0.018 |
| Wave 3 | 0.98 (0.88, 1.08) | 0.66 | 0.78 (0.68, 0.89) | <0.001 |
| Wave 4 | 0.93 (0.78, 1.10) | 0.41 | 0.81 (0.65, 1.02) | 0.068 |
| Wave 5 | 1.92 (1.69, 2.18) | <0.001 | 0.76 (0.62, 0.92) | 0.006 |
| Income quartiles |  |  |  |  |
| Q4 | Reference |  | Reference |  |
| Q1 | 1.73 (1.54, 1.95) | <0.001 | 1.20 (1.03, 1.40) | 0.020 |
| Q2 | 2.24 (2.00, 2.51) | <0.001 | 1.21 (1.06, 1.40) | 0.006 |
| Q3 | 1.48 (1.31, 1.66) | <0.001 | 1.15 (1.01, 1.32) | 0.040 |
| Education level |  |  |  |  |
| >12 years | Reference |  | Reference |  |
| <9 years | 1.72 (1.53, 1.93) | <0.001 | 1.11 (0.96, 1.27) | 0.15 |
| 10-12 years | 1.04 (0.94, 1.15) | 0.42 | 1.01 (0.90, 1.14) | 0.81 |
| 9 years | 1.01 (0.88, 1.16) | 0.87 | 1.03 (0.87, 1.20) | 0.76 |
| Country-Of-Origin |  |  |  |  |
| Nordic | Reference |  | Reference |  |
| European | 0.78 (0.69, 0.89) | <0.001 | 1.01 (0.87, 1.18) | 0.86 |
| Other | 0.72 (0.66, 0.80) | <0.001 | 1.13 (0.99, 1.29) | 0.067 |
| Household size |  |  |  |  |
| Single-person household | Reference |  | Reference |  |
| Multi-person household | 0.69 (0.64, 0.75) | <0.001 | 0.90 (0.82, 0.99) | 0.038 |
| Marital status |  |  |  |  |
| No registered partner | Reference |  |  |  |
| Registered partner | 1.03 (0.95, 1.12) | 0.42 |  |  |
| Population density |  |  |  |  |
| Rural | Reference |  |  |  |
| Urban | 1.11 (0.97, 1.28) | 0.12 |  |  |
