# Supplementary material for: Impact of socioeconomic status and country of origin on COVID-19 outcomes in Swedish ICUs: a retrospective registry-based cohort study
Source: BMJ Open. 2025 Nov 16;15(11):e099763. doi: 10.1136/bmjopen-2025-099763 (PMC12625895; doi:10.1136/bmjopen-2025-099763)
Supplement: Supplementary file 2 [file bmjopen-2025-099763.reviewer_comments.pdf]

PEER REVIEW HISTORY

BMJ Open publishes all reviews undertaken for accepted manuscripts. Reviewers are asked to complete a checklist review form and are provided with free text boxes to elaborate on their assessment. These free text comments are reproduced below.

ARTICLE DETAILS

Title (Provisional)

Impact of Socioeconomic Status and Country-of-Origin on COVID-19 Outcomes in Swedish ICUs: A Retrospective Registry-Based Cohort Study

Authors

Taxbro, Knut; Åhman, Rasmus; Chew, Michelle S.; Engerström, Lars

---

VERSION 1 - REVIEW

**Reviewer**	**1**
**Name**	**Termorshuizen, Fabian**
**Affiliation**	**Amsterdam UMC Locatie AMC, Department of Medical Informatics**
**Date**	**20-May-2025**
**COI**	**None**

Review

This is an important register-based study on the influence of socio-economic status and country of origin on mortality following ICU admission for COVID-19 infection, resulting in a worth reading manuscript.

Many important variables were registered including details on comorbidity, income and vaccination status, which makes that these data give an unique opportunity to answer many important questions concerning the epidemiology of COVID-19.

However, I have some major questions concerning the analysis and presentation of the data. Please, find my comments and suggestions for improvement in the attached word document.

Introduction

'… Specifically, being born in Africa and lower income level were associated with an increased 90-day mortality …'

The question is whether this is due to higher infection rates in these groups or worse prognosis after hospital admission. It is not clear why these findings challenge the concept of equitable health care.
Even in case of equitable health care there may be patient-related factors such as comoborbity, or lifestyle characteristics that may cause a worse prognosis in disadvantaged groups.

Methods

'… Patients with incomplete 90-day mortality data were excluded from the final analysis. …'

How many patients were excluded for this reason and why not including them as censored observations in a survival analysis?

Statistical analysis

Figure 2 shows a Kaplan Meier Survival curve. Why not analyse the data with a Cox regression analysis in stead of a logistic regression model.

Why not breaking down the category 'Other' in country of origin in those of 'Western'vs those of 'non-Western' origin, especially in view of the findings mentioned in the introduction that those from African origin belong to the most vulnerable.

It is not clear what is the outcome in the analysis on renal replacement therapy (RRT) during the first 60 days. Is death or start of therapy the outcome? And censoring at 60 days? Why not analyse such data in a parametric or non-parametric survival model? Duration to a certain event is often not Normally distributed, and thus, techniques for survival analysis are better suited. Under 'Study objectives' both ventilator and RRT free days are mentioned as secondar outcome.

Results

Table 1 (Uni- and multivariable regression)
Income quartiles: the individual P values do not suggest a strong statistically significant effect. However, one P value is very close to 0.05 (0.051). Testing the three estimated parameters (Q1,Q2, Q3 vs Q4) in one step in a post-estimation Wald-X2 (df=3) may demonstrate statistical significance. In general, cluster-wise testing of the estimates that associated with one variable (here: Income) is better than evaluating separate effect estimates. I feel that the conclusion that '… socioeconomic variables did not significantly impact mortality…' is not supported by the data.

It is a remarkable finding that in univariable analysis originating from a non-European country has such a low OR (0.75 [0.67-0.84]). As migrants are often in disadvantaged position and has on average a lower income and lower income is associated with higher mortality (1.77 [1.54-2.04]) you may expect an OR >1.00 in the univariate analysis. Adjustment for what factor did cause the reversing of the OR? Maybe a divergent age distribution?

As ethnic origin and socio-economic status are the variables of main interest, the authors may consider to present descriptive data by country of origin and income categories. The Tables 1 and 2 are important from a overall general point of view, but not acuminate to the research question.

What is the reason that only 5833 patients out of the 8025 patients are included in the multivariable models? The step from 8025 to 5833 should be described in the flow chart of Figure 1.

The E-value needs some clarification for those readers not familiar with this statistic.

Discussion

I feel that the conclusion that the results challenge the notion of equitable health care is not supported by the data. There may be important patient-related differences such as life style factors or the severity of pre-existing comorbidities that may be associated with more vulnerability and higher mortality risks, even in case of equitable health care.
Furthermore, an inconsistency in the line of reasoning is that the authors mention the 'health and a right to health care is mandated by law in Sweden and the funding principles of healthcare' as reason for not finding a clear effect of socio-economic variables on mortality (page 13, line 20). So for what reason are possible gaps in the publicly funded and universal coverage now an explanation for the higher mortality rates among those from non-European countries?

**Reviewer**	**2**
**Name**	**Mullany, Dan**
**Affiliation**	**The Prince Charles Hospital, Anaesthesia and Perfusion Services**
**Date**	**21-Aug-2025**
**COI**	**None**

Summary

The abstract summarises the study, methods and results well

The authors studied the association between socioeconomic variables, country of origin and outcomes in adults with acute hypoxaemic respiratory failure secondary to COVID- 19 admitted to an ICU in Sweden. The primary outcome was 90 day mortality and secondary outcomes were 1 year mortality, ventilator and renal replacement therapy free days within 60 days post ICU admission. The authors used individual level data from the Swedish Intensive Care registry and Statistics Sweden.

Originality

There is a large body of work on the association of measures of SES and outcomes in critically ill adults. Generally, but not universally, lower SES is associated with worse health outcomes. The original element of this study is the examination of the patient's country of

origin in a high-income country with universal healthcare. The association between race, SES disadvantage and worse health outcomes is well established. The authors also had access to individual measures of SES rather than area level measures. The authors also provided a near complete nationwide scope during the peak of COVID-19 pandemic.

Importance

Evidence-based medicine

The evidence is presented scientifically and is easy to follow

Research question

The research question is clearly presented and answered

Study design

The study design is adequate.

Participants

The participants are clearly identified and described

Methods

In the methods, I have some questions:

-How was missing data dealt with in the analysis? There are 8025 patients in Table 1 but 5833 (73%) in the regression model (page 23). Were independent variables as well as outcomes missing?

-Swedish PIN; would all non-European country of origin (study) patients have a PIN?

-Would surviving “Other Country“ patients be more likely to be lost to follow up compared to Swedish nationals?

-How were the multiple ICU admissions and transfers dealt with in the model?

-Does 90 day mortality include all-cause mortality both in and out of hospital?

-Were interaction terms considered in the models?

-Were the properties/ adequacy/ assumptions of the statistical models (eg area under ROC, calibration, proportional hazards etc) evaluated?

-Was clustering at hospital level considered ? As I understand it, there are approximately 80 ICU’s in Sweden of varying sizes from the highly prestigious Karolinska Institute to smaller rural units.

-Was a more detailed sensitivity analysis (other than E value) of the complete case analysis considered?

- Was there consideration of causal inference methods and treatment effects analysis?

Results

“Other” country of origin patients had a lower 90 day crude mortality than the other 2 groups. There is a marked change in the odds ratio for the Country of origin from the univariable to the multivariable analysis. “Other Country”= 0.75(0.67-0.84) in the univariable but changes to 1.34 (1.13-1.61) in the multivariable analysis. This means that the conclusions are critically dependent on the statistical model and risk adjustment. Important confounder(s) that markedly change the direction of the effect of country of origin must be present.

Would the authors consider a table comparing demographic, clinical factors and treatments for the main study variable (Country of Origin as the heading)? That may allow the reader to better understand potential confounding factors. E.g Were “Other country” of origin patients: less likely to be vaccinated, more critically ill, referred to ICU later, receiving the same intensity of treatment ?

Don’t need SM tables 1 and 2, It is covered in Table 1.

There are minor discrepancies in the numbers. There are 5833 included in Table 1(page 23) but 3068 in SM Table 1 (age <65y) and 2770 in SM Table 2 (age >=65y) for a total of 5838 and 5830 included in Figure 2.

Is there a Cox model for Figure 2?

“Vasoactive drugs” has high percent of missing and is not included in the model but is likely to be a significant confounder.

Interpretation and conclusions

These are clear and well written.

Income, education, occupation and other factors have a varying influence on SES depending on the age of patient. A university student from a wealthy family may have a similar income to an unskilled worker. Elderly patients may have a low income but significant accumulated assets. The SES of the area in which the patient lives may be influential on health outcomes.

Would the authors consider further discussion on the causal pathway from country of origin to 90 day mortality. Some confounders eg severiy of illness may be due direclty of country of origin and which confounders are appropriate to include in the risk adjustment.

References

Are up to date and relevant

Abstract/summary/key messages

These are well written and clear

Reporting standards

Compliance with STROBE is not stated.

VERSION 1 - AUTHOR RESPONSE

Reviewer: 1

Dr. Fabian Termorshuizen, Amsterdam UMC Locatie AMC

Comments to the Author:

Review

This is an important register-based study on the influence of socio-economic status and country of origin on mortality following ICU admission for COVID-19 infection, resulting in a worth reading manuscript.

Many important variables were registered including details on comorbidity, income and vaccination status, which makes that these data give an unique opportunity to answer many important questions concerning the epidemiology of COVID-19.

However, I have some major questions concerning the analysis and presentation of the data. Please, find my comments and suggestions for improvement in the attached word document.

Attachment:

Introduction

'… Specifically, being born in Africa and lower income level were associated with an increased 90-

day mortality …'

The question is whether this is due to higher infection rates in these groups or worse prognosis after hospital admission. It is not clear why these findings challenge the concept of equitable health care.

Even in case of equitable health care there may be patient-related factors such as comoborbity, or lifestyle characteristics that may cause a worse prognosis in disadvantaged groups.

Response: Thank you for this insightful remark. We have revised the second last paragraph of the introduction.

Methods

'… Patients with incomplete 90-day mortality data were excluded from the final analysis. …'

How many patients were excluded for this reason and why not including them as censored

observations in a survival analysis?

Response: Thank you for this excellent question. These 26 patients were excluded due to missing mortality data. They could therefore not be included in an analysis of mortality. This is now clarified in Flowchart (Fig. 1).

Statistical analysis

Figure 2 shows a Kaplan Meier Survival curve. Why not analyse the data with a Cox regression

analysis instead of a logistic regression model.

Response: We appreciate the opportunity to clarify our statistical approach. Our study used both logistic regression and Cox regression analyses. The logistic regression was used to determine the independent association of predictor variables with the primary outcome (90-day mortality) and is presented in Table 3. We also used Cox regression to create survival plots (Figure 2) that visualize the association between country-of-origin and mortality over a one-year period. This dual approach allowed us to analyse both short-term mortality and long-term survival. We have added a cox model for visualisation which is now included in the supplement.

Why not breaking down the category 'Other' in country of origin in those of 'Western'vs those of 'non-Western' origin, especially in view of the findings mentioned in the introduction that those from African origin belong to the most vulnerable.

Response: Regarding the "Other" country-of-origin category, we utilized a classification based on the United Nations' standard for grouping countries. This approach, which includes Nordic, European, and a combined "Other" category, is well-established in similar research and was chosen to maintain consistency with existing literature. Also, the numbers of observations in the different countries making up the "other" group were quite low, and we wanted to prevent potential disclosure risk. While disaggregated data provides important nuanced information, such small sample sizes would not have enabled robust statistical analyses.

It is not clear what is the outcome in the analysis on renal replacement therapy (RRT) during the first 60 days. Is death or start of therapy the outcome? And censoring at 60 days?

Response: We have revised and clarified under "Study objectives". While we used 90-day mortality as our primary outcome, we also calculated the organ support free days at 60 days (secondary outcome). Most of the mortality as well as most of the organ support was within that time frame.

Why not analyse such data in a parametric or non-parametric survival model? Duration to a certain event is often not normally distributed, and thus, techniques for survival analysis are better suited.

Response: We agree to this statement, even though the results often are similar when comparing these different statistical methods (https://bmcmedresmethodol.biomedcentral.com/articles/10.1186/s12874-023-01963-z .

We agree that the organ support free days is not normally distributed and that it is therefore more accurate to model it with another model than linear regression. We have replaced the linear regression with an ordinal logistic model which we think is more appropriate. In this case it affected the significance of some of the socioeconomic factors.

Organ support free days was analysed using ordinal logistic regression.

Organ support free days, CRRT-free days and ventilator free days were statistically significantly reduced for "other country" and lower income quartiles similar to the linear regression.

Under 'Study objectives' both ventilator and RRT free days are mentioned as secondary outcome.

Response: We apologize for being unclear: Ventilator free days and RRT free days (separately and in combination) were both secondary outcomes, as was 1 year mortality. We believe that more than one secondary outcome is acceptable. (Anesth Analg. 2017 Aug;125(2):678-681.

doi: 10.1213/ANE.0000000000002224.) If we have not interpreted this comment correctly, we welcome clarification and will be happy to revise accordingly.

Results

Table 1 (Uni- and multivariable regression)

Income quartiles: the individual P values do not suggest a strong statistically significant effect.

However, one P value is very close to 0.05 (0.051). Testing the three estimated parameters (Q1,Q2,

Q3 vs Q4) in one step in a post-estimation Wald-X2 (df=3) may demonstrate statistical significance.

In general, cluster-wise testing of the estimates that associated with one variable (here: Income) is better than evaluating separate effect estimates. I feel that the conclusion that '… socioeconomic variables did not significantly impact mortality…' is not supported by the data.

Response: We thank the reviewer for this insightful comment. In table 1 the global p-values for categorical variables are calculated using likelihood ratio tests which we forgot to mention. We have now added "Global p-values for categorical variables were calculated using likelihood ratio tests".

"Socioeconomic variables, including income quartiles, did not show a statistically significant association with 90-day mortality in the multivariable model, and neither after analysing the joint effect of income quartiles Wald Chi2 =4.3, p= 0.23 " The result of likelihood ratio test (p=0.25) is similar to Wald Chi2 =4.3, p= 0.23 for the joint effect of income quartiles on 90-day mortality in the multivariable model.

It is a remarkable finding that in univariable analysis originating from a non-European country has such a low OR (0.75 [0.67-0.84]). As migrants are often in disadvantaged position and has on

average a lower income and lower income is associated with higher mortality (1.77 [1.54-2.04]) you

may expect an OR >1.00 in the univariate analysis. Adjustment for what factor did cause the

reversing of the OR? Maybe a divergent age distribution?

Response: Yes, the reviewer is entirely correct in his hypothesis regarding age; the reversal of the OR was due to the age adjustment

As ethnic origin and socio-economic status are the variables of main interest, the authors may

consider to present descriptive data by country of origin and income categories. The Tables 1 and 2 are important from a overall general point of view, but not acuminate to the research question.

Response: We fully agree with this comment and have added another table (table 4) showing baseline data across county-of-origin and income.

What is the reason that only 5833 patients out of the 8025 patients are included in the multivariable

models? The step from 8025 to 5833 should be described in the flow chart of Figure 1.

Response: Thank you for your comment regarding this key feature. We have added an additional box in Figure 1 to help clarify the missing data.

The E-value needs some clarification for those readers not familiar with this statistic.

Response: We have added clarification regarding E-value under methods.

Discussion

I feel that the conclusion that the results challenge the notion of equitable health care is not

supported by the data. There may be important patient-related differences such as lifestyle factors or the severity of pre-existing comorbidities that may be associated with more vulnerability and higher mortality risks, even in case of equitable health care.

Furthermore, an inconsistency in the line of reasoning is that the authors mention the 'health and a right to health care is mandated by law in Sweden and the funding principles of healthcare' as reason for not finding a clear effect of socio-economic variables on mortality (page 13, line 20). So for what reason are possible gaps in the publicly funded and universal coverage now an explanation for the higher mortality rates among those from non-European countries?

Response: We acknowledge this remark and have revised the conclusion accordingly, aiming to present a more cautious interpretation and to avoid overstating implications regarding equitable health care. One important lesson learnt from the OpenSAFELY database in the UK is that disaggregated data is essential for understanding heterogeneity of outcome effects. Unfortunately, we were unable to provide more nuance to this beyond classifying country of origin into 3 categories. The 'other' category very likely represents a wide range of different populations with varying socioeconomic indicators. As the numbers of patients within each country of origin in this category were very low, we were obliged to pool this data in order to avoid inadvertent risk of re-identifying individuals in small subgroups.

.

Reviewer: 2
Dr. Dan Mullany, The Prince Charles Hospital, St Vincent's Private Hospital Northside
Comments to the Author:
Summary
The abstract summarises the study, methods and results well
The authors studied the association between socioeconomic variables, country of origin and outcomes in adults with acute hypoxaemic respiratory failure secondary to COVID- 19 admitted to an ICU in Sweden. The primary outcome was 90 day mortality and secondary outcomes were 1 year mortality, ventilator and renal replacement therapy free days within 60 days post ICU admission. The authors used individual level data from the Swedish Intensive Care registry and Statistics Sweden.

Originality
There is a large body of work on the association of measures of SES and outcomes in critically ill adults. Generally, but not universally, lower SES is associated with worse health outcomes. The original element of this study is the examination of the patient's country of origin in a high-income country with universal healthcare. The association between race, SES disadvantage and worse health outcomes is well established. The authors also had access to individual measures of SES rather than area level measures. The authors also provided a near complete nationwide scope during the peak of COVID-19 pandemic.

Importance
Evidence-based medicine
The evidence is presented scientifically and is easy to follow

Research question
The research question is clearly presented and answered
Study design
The study design is adequate.
Participants
The participants are clearly identified and described
Methods
In the methods, I have some questions:

-How was missing data dealt with in the analysis? There are 8025 patients in Table 1 but 5833 (73%) in the regression model (page 23). Were independent variables as well as outcomes missing?

Response: Thank you for this comment, which is similar to a comment made by Reviewer 1.We have now clarified how missing data was handled in different levels of analysis, see Figure 1.

-Swedish PIN; would all non-European country of origin (study) patients have a PIN?

Response: Yes, we confirm that all patients included in the study have a Swedish personal identification number.

-Would surviving "Other Country" patients be more likely to be lost to follow up compared to Swedish nationals?

Response: Very few patients were lost to follow up within 90 days (26 of 8051 patients), 2415 did not survive for 90 days, so the ones lost to follow up were around 1% of the number of deaths. There was no indication that non-Europeans were over-represented among these patients, which is a major strength in Swedish registry-based research (REF: https://doi.org/10.1186/1471-2458-11-450)

-How were the multiple ICU admissions and transfers dealt with in the model?

Response: The analysis is conducted on a patient level basis. The times (90-day mortality, symptoms to ICU, hospital admission to ICU) are all calculated from the first ICU admission while all care is summarised across all ICU-admissions for each patient. 90-day mortality includes all-cause and -location mortality. We have now clarified this in the methods section.

-Does 90 day mortality include all-cause mortality both in and out of hospital?

Response: Yes. We have now clarified this accordingly.

-Were interaction terms considered in the models?

Response: The interactions considered are reported on page 10. These were selected based on current available evidence and clinical plausibility.

-Were the properties/ adequacy/ assumptions of the statistical models (eg area under ROC, calibration, proportional hazards etc) evaluated?

Response: Thank you for this relevant comment. For the main model, the AUC ROC was 0.784 (95% CI: 0.771-0.796) Hosmer-Lemeshow goodness of fit test did not show a significant deviation from perfect calibration in 10 equally sized groups, p=0.75. (Table 3). We acknowledge the importance of evaluating model adequacy in multivariable logistic regression. A commonly used rule of thumb recommends a minimum of 10 outcome events per variable (EPV) included in the model to ensure stability and reduce the risk of overfitting (Peduzzi et al., 1996). In our cohort, with 2,415 deaths among 8,025 ICU-treated COVID-19 patients and having 23 variables included in our multivariable model (Table 3), we exceed the recommended EPV threshold by a wide margin. We therefore consider our models to be well-powered and statistically appropriate in terms of variable-to-event ratio.

The Cox model assumptions of proportional hazard was evaluated using shoenfield residuals, graphically and by testing for correlation between them and survival time. (The assumption of prop hazards was not met) - we have clarified this in the table legends (Supplement 11)

-Was clustering at hospital level considered ? As I understand it, there are approximately 80 ICU's in Sweden of varying sizes from the highly prestigious Karolinska Institute to smaller rural units.

Response: Thank you for this insightful comment. There has been considerable debate in Sweden regarding this topic. Although we believe that the treatments do not vary to a great deal between hospitals, it is possible that the severity of disease required to be admitted to the ICU varies between hospitals. We have added a general estimating equation logistic regression model to allow for clustering on index admission hospital. In this model income quartiles are also statistically significant. This model is now included in the supplementary material as Supplement 10.

-Was a more detailed sensitivity analysis (other than E value) of the complete case analysis considered?

Response: The sensitivity analyses made are presented in supplementary tables 1-3 (age groups and on IMV). These were selected based on current evidence from previous research on similar cohorts. Also, based on reviewer feedback, we have performed a sensitivity analysis in the form a general estimating equation logistic regression model to allow for clustering on index admission hospital (supplement 10). We did not identify a need for any further sensitivity analyses apart from these.

- Was there consideration of causal inference methods and treatment effects analysis?

Response: We thank the reviewer for this suggestion. Given the novel nature of COVID-19 in the ICU and the limited prior evidence regarding socioeconomic factors, we felt that attempting formal causal inference (eg. using causal diagrams or mediation models) would be too speculative. Our study was designed to describe associations and patterns in the data rather than to estimate causal effects. We therefore focused on multivariable regression analyses, adjusting for potential covariates on an equal basis. In addition, a more detailed analysis of treatment effects was not the objective in this study. However, we acknowledge that future studies incorporating causal inference or treatment effect analyses could provide valuable insights on this topic.

Results
"Other" country of origin patients had a lower 90 day crude mortality than the other 2 groups. There is a marked change in the odds ratio for the Country of origin from the univariable to the multivariable analysis. "Other Country"= 0.75(0.67-0.84) in the univariable but changes to 1.34 (1.13-1.61) in the multivariable analysis. This means that the conclusions are critically dependent on the statistical model and risk adjustment. Important confounder(s) that markedly change the direction of the effect of country of origin must be present.
Would the authors consider a table comparing demographic, clinical factors and treatments for the main study variable (Country of Origin as the heading)? That may allow the reader to better understand potential confounding factors. E.g Were "Other country" of origin patients: less likely to be vaccinated, more critically ill, referred to ICU later, receiving the same intensity of treatment ?

Response: This comment is similar to a comment from reviewer 1 and we have now added a new table (table 4) showing the requested data across country of origin.

Don't need SM tables 1 and 2, It is covered in Table 1.

Response: The supplementary Tables 1 and 2 are supposed to present distinct features of young and older patients, and we believe they add relevant information for the sensitivity analysis. However, we would be happy to revise this according to the reviewer's/editor´s preferences.

There are minor discrepancies in the numbers. There are 5833 included in Table 1(page 23) but 3068 in SM Table 1 (age <65y) and 2770 in SM Table 2 (age >=65y) for a total of 5838 and 5830 included in Figure 2.

Response: Thank you, we are very grateful for identifying problems with the number of patients included in the regression analyses! We have included as many patients as possible in the multivariable analyses. Depending on missing data and differences in variables included in the multivariable models, the numbers differ. We have tried to clarify this by describing the number of patients with complete data and the number of patients in the multivariable models. We have also added a description of the number of missing data for the variables in the models.

Is there a Cox model for Figure 2?

Response: Thank you for this remark. We have added a table for the Cox survival curve in the supplementary material 11.

"Vasoactive drugs" has high percent of missing and is not included in the model but is likely to be a significant confounder.

Response: We thank the reviewer for this important point. We agree that the use of vasoactive drugs is a key indicator of illness severity and thus a potential major confounder in the relationship between socioeconomic factors and patient outcomes. As the reviewer correctly notes, this variable could not be included in our primary model due to a high percentage of missing data. However, we believe this unmeasured confounding may be partially mitigated by the inclusion of other metrics describing disease severity, such as SAPS3 score, level of respiratory support and degree of ARDS.

Interpretation and conclusions
These are clear and well written.
Income, education, occupation and other factors have a varying influence on SES depending on the age of patient. A university student from a wealthy family may have a similar income to an unskilled worker. Elderly patients may have a low income but significant accumulated assets. The SES of the area in which the patient lives may be influential on health outcomes. Would the authors consider further discussion on the causal pathway from country of origin to 90 day mortality. Some confounders eg severiy of illness may be due direclty of country of origin and which confounders are appropriate to include in the risk adjustment.

Response: The reviewer points out some highly relevant potential confounders that we could not include in our analysis. Based on this, we have revised the section discussing limitations, as well as the conclusion of the current study.

References
Are up to date and relevant
Abstract/summary/key messages
These are well written and clear

Reporting standards
Compliance with STROBE is not stated.

Response: We have added a sentence on STROBE compliance under methods.

Reviewer: 1
If you have selected 'Yes' above, please provide details of any competing interests.: Not applicable

Reviewer: 2
If you have selected 'Yes' above, please provide details of any competing interests.: I have previously met Prof Chew through the CCRG in Brisbane Australia.

VERSION 2 - REVIEW

**Reviewer**	**1**
**Name**	**Termorshuizen, Fabian**
**Affiliation**	**Amsterdam UMC Locatie AMC, Department of Medical Informatics**
**Date**	**21-Oct-2025**
**COI**	

The authors did a lot of work to improve the manuscript and addressed the raised issues adequately. One comment: I suggested to further categorize the 'other countries' in Western and non-Western, not to report for individual countries of origin separately. Thus, still in broad categories. I wonder whether this might be possible without disclosure risk. And may be a suggestion for future studies.

**Reviewer**	**2**
**Name**	**Mullany, Dan**
**Affiliation**	**The Prince Charles Hospital, Anaesthesia and Perfusion Services**

Date **25-Oct-2025**

COI

Issues raised have been addressed.
